# Enhancing the anticancer potential of garlic and lemon essential oils through nanoemulsification: a comparative study

**DOI:** 10.1038/s41598-026-65811-y

**Published:** 2026-08-21

**Authors:** Eman M. Elgendy, Lobna Shelbaya, Eman M. Ramadan, Ashraf R. El-Zainy

**Affiliations:** Department of Home Economy, Faculty of Specific Education, Mansoura University, Mansoura, Egypt

**Keywords:** Antioxidant, Essential oils, Garlic, Lemon, Nanoemulsion, Biochemistry, Biotechnology, Cancer, Chemistry, Nanoscience and technology, Plant sciences

## Abstract

Garlic and lemon essential oils are recognized for their health benefits, and this study analyzed their chemical composition using gas chromatography-mass spectrometry. This study emphasized reducing these oils to nanoemulsion forms. Both conventional and nanoemulsified oils were assessed for their effects on breast cancer cells (MCF-7). Results indicated that both normal and nano garlic essential oils exhibited a pronounced anticancer effect, resulting in lower cell viability. In contrast, lemon essential oils showed moderate efficacy with higher cell viability. The DPPH assay demonstrated that nano garlic essential oil had the strongest antioxidant capacity, while also preserving acid and peroxide values better than traditional forms. Lemon essential oil displayed some effectiveness but was less potent overall. The study concludes that reducing garlic and lemon essential oils to the nanoscale enhances their efficacy, particularly highlighting garlic’s volatile oils as a potentially valuable remedy for human health compared with lemon’s.

## Introduction

Plants used for both food and medicine contribute significantly to nutrition and wellness. Their bioactive constituents demonstrate immune-modulating, antioxidant, anti-inflammatory, and antimicrobial effects^1,2^.

Essential oils (EOs) are fragrant compounds extracted from various parts of plants. They comprise a complex mixture of volatile and biologically active organic substances. Typically, essential oils appear as transparent, pale yellow liquids with a density lower than water. When exposed to heat and light, these oils are particularly susceptible to oxidation due to reactive functional groups, such as hydroxyl and olefinic double bonds^3^. Essential oils exhibit effective antibacterial and antioxidant properties, making them a potential source of natural compounds suitable for food applications^4^. Essential oils are acknowledged in conventional medicine for their capacity to act as medicinal agents, suggesting they can treat or avert different ailments. Essential oils derived from plants contain a variety of volatile bioactive phytochemicals, such as phenolics, flavonoids, and terpenes^5^.

Garlic has historically been appreciated as a key ingredient in cooking and a traditional medicinal herb, primarily due to its rich content of organosulfur compounds. Recent research has highlighted the significant medicinal value of garlic, demonstrating its powerful immunomodulatory, anti-inflammatory, and antioxidant effects. The biological effects are significantly influenced by how garlic is used—whether raw, cooked, or in extract form—emphasising the importance of preparation techniques in optimising its health benefits^6^.

Lemon essential oil (LEO) is usually extracted from lemon peels by means of steam distillation. It comprises more than two hundred parts. These bioactive substances are recognized for their extensive health-enhancing benefits, such as antioxidant, anticancer, antiviral, anti-inflammatory, and antibacterial effects^7^.

Combining nanomedicine with traditional plant-based products enhances the solubility, stability, and targeted delivery of bioactive compounds, bridging traditional healing with modern science to develop safer, more effective therapeutics^8–10^.

Nanoemulsions are pharmaceutical formulations consisting of nanoparticles in the nanoscale range. The creation of nanoemulsions for the delivery of functional chemicals is a recent application of nanotechnology.

Numerous researchers have examined multifunctional nanoemulsions in diverse areas^11,12^.

These fields primarily aim to help treat different types of cancer. The study demonstrated that tumour cells easily absorb nanoemulsions, which inhibit tumour growth, are non-toxic to healthy cells, and block the spread of cancer cells^13^.

Essential oils in nanoemulsions have emerged as an effective strategy to enhance their efficacy, stability, and practicality^14^. The unstable nature and poor water solubility of essential oils have greatly limited their practical use and effectiveness. To overcome these challenges, one of the most effective strategies is incorporating essential oils into nanoparticle-based delivery systems, which helps improve their stability and enhance their therapeutic potential^15^.

Cancer is an intricate, multi-dimensional illness marked by unchecked cell proliferation, evasion of apoptosis, and spread to other parts. The rates of incidence and mortality are increasing worldwide, especially in developing nations, attributed to elements such as pollution, inactive lifestyles, and infections. These elements can harm oncogenes, resulting in the onset of cancer^16^.

Due to the valuable properties of essential oils found in garlic and lemon, their use has been linked to improved overall performance, enhanced nutrient absorption, and increased digestive enzyme activity. Additionally, these oils contribute to stronger immunity, better antioxidant status, healthier lipid profiles, balanced gut microbiota, and reduced impact of heat stress^17^.

So, this study explores the essential oils of garlic and lemon, comparing their traditional forms with their nanoemulsified versions. It also examines their physical and chemical characteristics, with a focus on evaluating their effectiveness as natural antioxidants.

This is a prelude to further studies on the use and comparison of these oils in their regular and nano forms as antimicrobial, antifungal, and anticancer agents, and their application as preservatives in some food products.

## Results and discussion

### Results

#### Fractionation and characterization of essential oil compounds using GC/MS

GC/MS extensively analyzes terpenes, fatty acids, esters, aldehydes, and alcohols.

This outcome highlights the identification of the chemical constituents in the volatile oils of garlic and lemon through Gas chromatography–mass spectrometry analysis.

##### Identification and characterization of chemical components in garlic essential oil

**Fig. 1:**
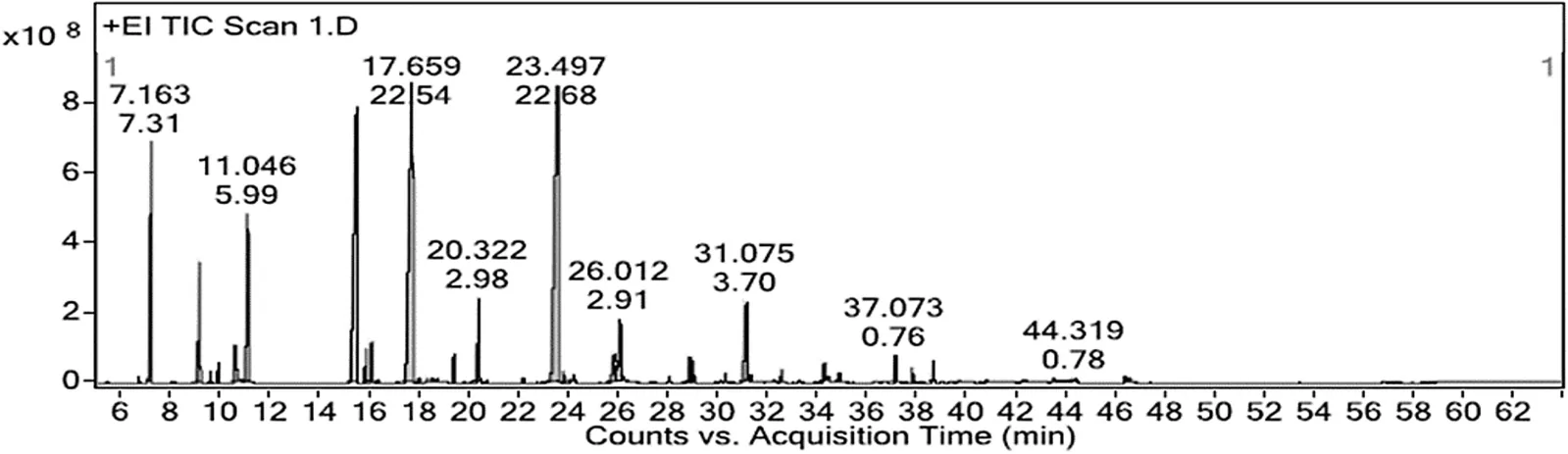
fractionation and identification of chemical components of garlic essential oil by Gas chromatography/mass spectrometry analysis.

**Fig. 2:**
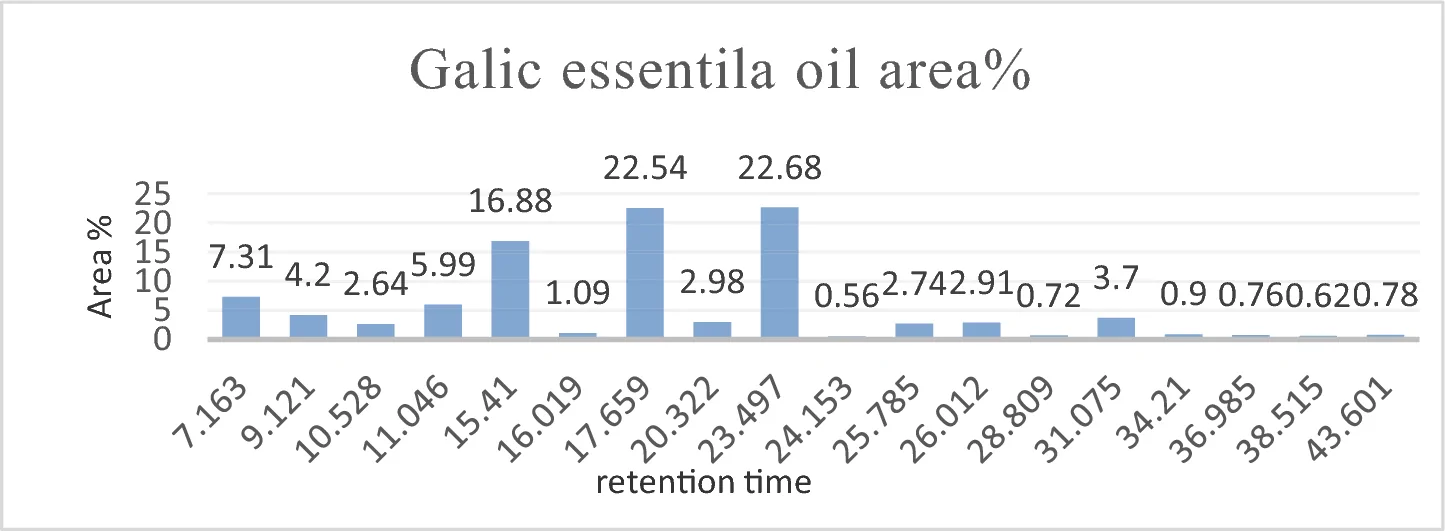
the area % for chemical constituents of garlic essential oil (GEO).

**Fig. 3:**
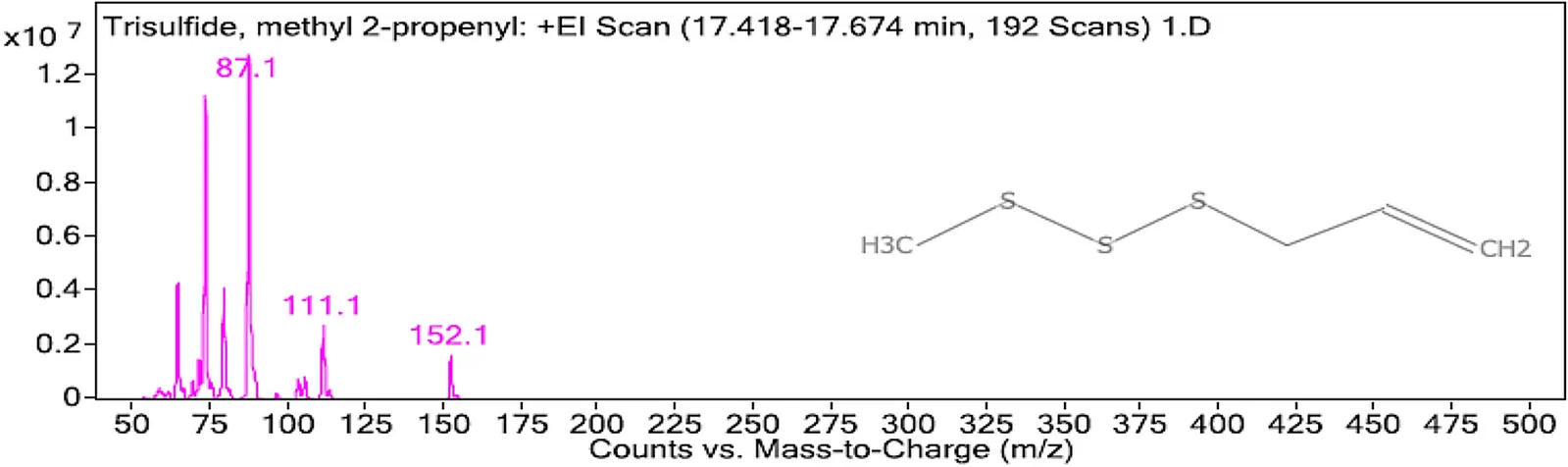
Fragmentation diagram of Trisulfide, methyl 2-propenyl (the major component of GEO).

**Fig. 4:**
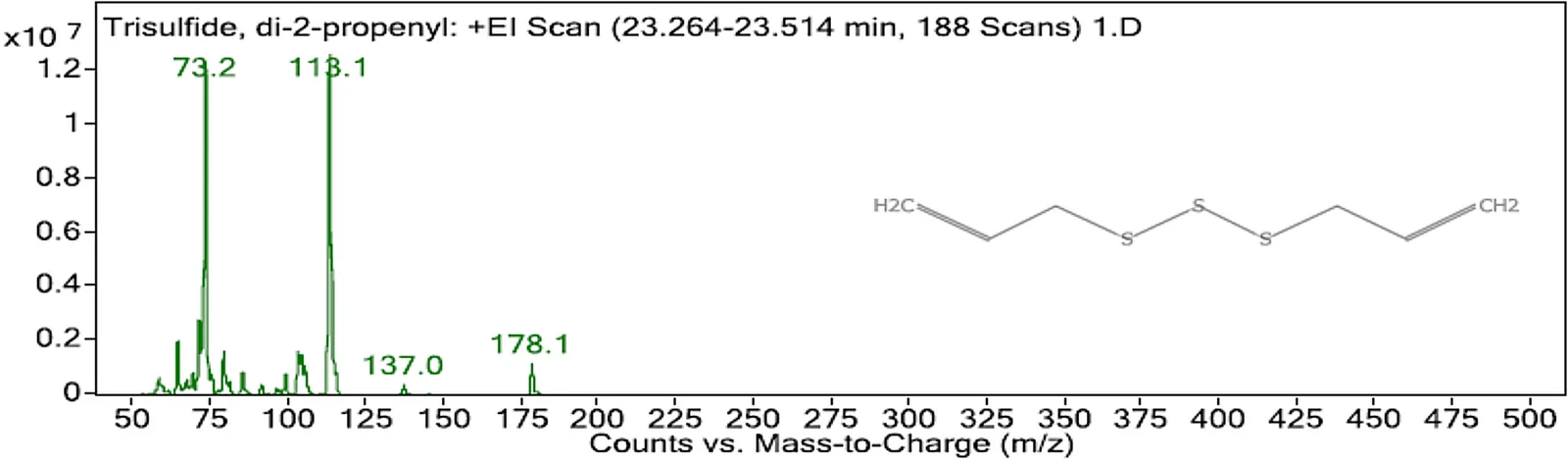
Fragmentation diagram of Trisulfide, di-2-propenyl (the major component of GEO).

Table 1 and Figs. 1, 2, 3, 4 display the findings from the GC/MS analysis of garlic essential oil. This analysis revealed that the garlic oil used in this study contained 18 components.

**Table 1:** Identification and characterization of chemical components in garlic volatile oil via GC/MS

Peak	Retention time	Component	Area %
1	43.601	1,2,3,4,5,6,7,8-Octathiocane	0.78
2	38.515	1-Allyl-3-(2-(allylthio)propyl) trisulfane	0.62
3	36.985	5-Methylthiophen-3-ylamine	0.76
4	34.21	4-Methyl-1,2,3-trithiolane	0.9
5	31.075	2-Ethylidene [1,3] dithiane	3.7
6	28.809	1-[2-Deoxy-.beta.-d-erythro-pentofurano syl] pyrrole-2,4-bisthiocarboxamide	0.72
7	26.012	2-(1-Cyano-3-methyl-but-2-phenyl amino)-but-2-enedinitrile	2.91
8	25.785	5-Methyl-1,2,3,4-tetrathionate	2.74
9	24.153	(Z)-1-Allyl-3-(prop-1-en-1-yl) trisulfane	0.56
10	23.497	Trisulfide, di-2-propenyl	22.68
11	20.322	2-Vinyl-4H- 1,3-dithiine	2.98
12	17.659	Trisulfide, methyl 2-propenyl	22.54
13	16.019	2-Ethylidene [1,3] dithiane	1.09
14	15.41	Diallyl disulphide	16.88
15	11.046	Dimethyl trisulfide	5.99
16	10.528	3H-1,2-Dithiole	2.64
17	9.121	Benzenamine, *N*, 4-dimethyl-	4.2
18	7.163	Diallyl sulfide	7.31

###### Identification and characterization of chemical components in lemon volatile oil

**Fig. 5:**
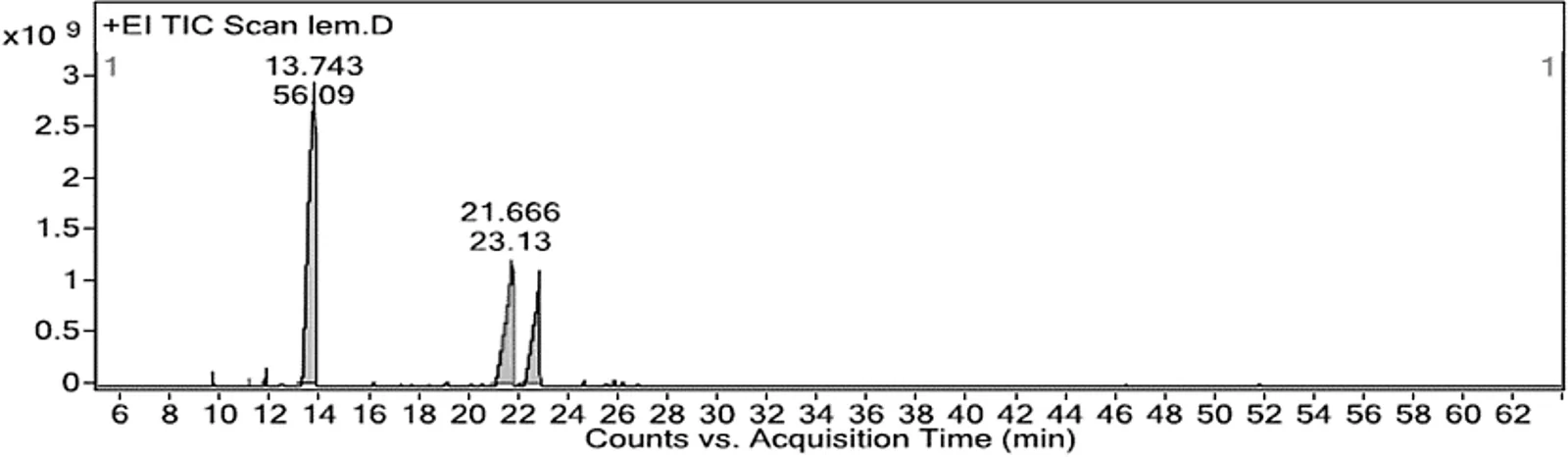
fractionation and identification of chemical components of lemon essential oil by GC/MS.

**Fig. 6:**
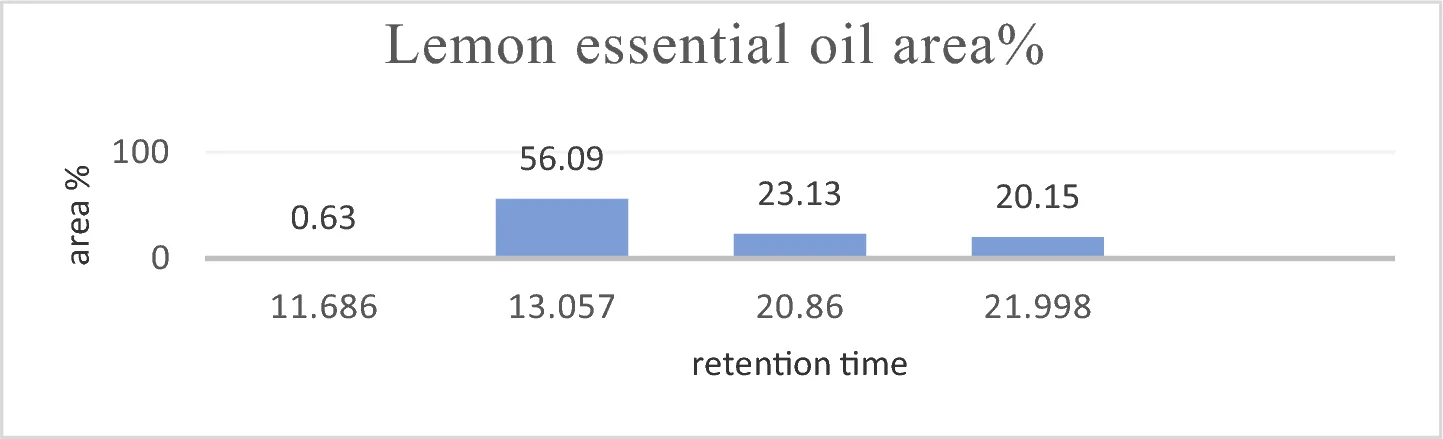
the area% % for chemical constituents of lemon essential oil (LEO).

**Fig. 7:**
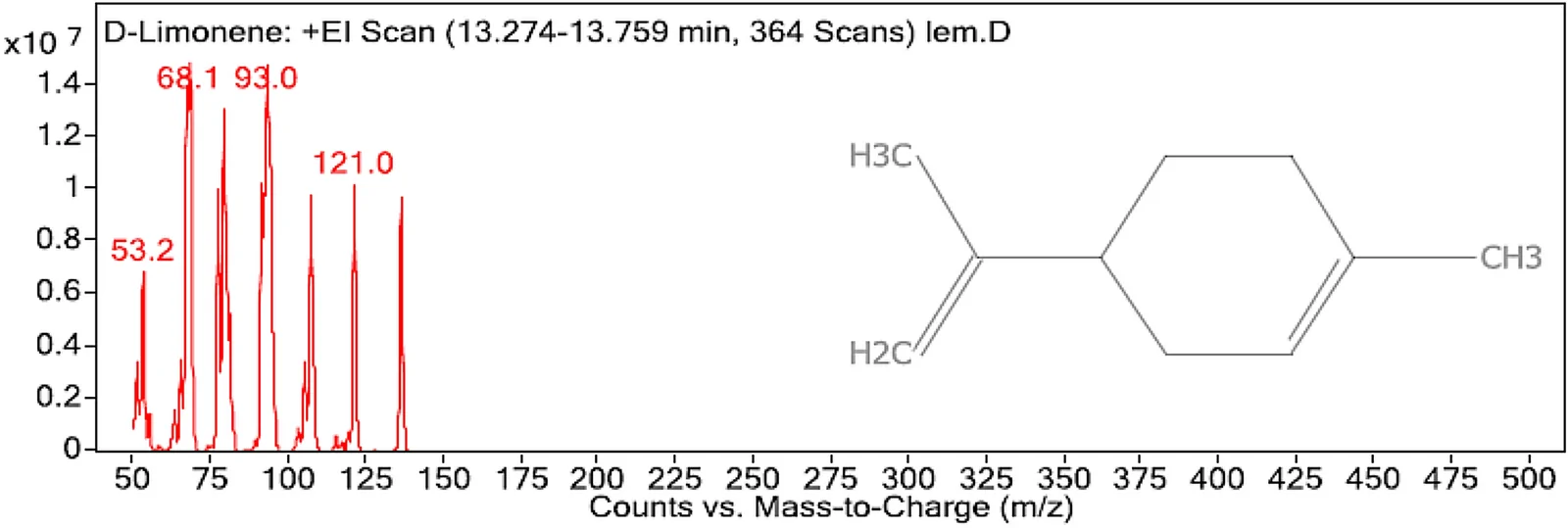
Fragmentation diagram of D-limonene (the major component of LEO).

The findings from the GC/MS analysis of lemon essential oil are presented in Table 2 and Figs. 5, 6, 7. These results reveal that the lemon essential oil analyzed in this study contains only the four main components that constitute the highest percentage of the essential oil, with D-limonene being the primary component at 56.09%.

**Table 2:** Fractionation and identification of chemical components of lemon essential oil by GC/MS

Peak	Retention time	Component	Area%
1	21.998	Citral	20.15
2	20.86	(1R)-cis- Verbenol	23.13
3	13.057	D-Limonene	56.09
4	11.686	Cyclohexane, 1-methylene-4-(1-methylethenyl)	0.63

 .

**Table 3:** Acid value of normal and nano volatile oils.

Oils	Normal	Nano	LSD _at 5%_
Lemon essential oil	7.18^a^ ± 0.065	6.35^b^ ± 0.060	0.02
Garlic essential oil	2.43^a^ ± 0.085	1.85^b^ ± 0.091	0.44

Significant values are different letters.

#### Characterization of volatile oils nano-emulsion

##### TEM (transmission electron microscopy)

Transmission electron microscopy (TEM) stands as a precise and accurate technique for nanoparticle characterisation, providing detailed visualisation of their morphology, dimensions, and dispersion.

###### TEM (transmission electron microscopy) of garlic volatile oil nanoemulsion

**Fig. 8:**
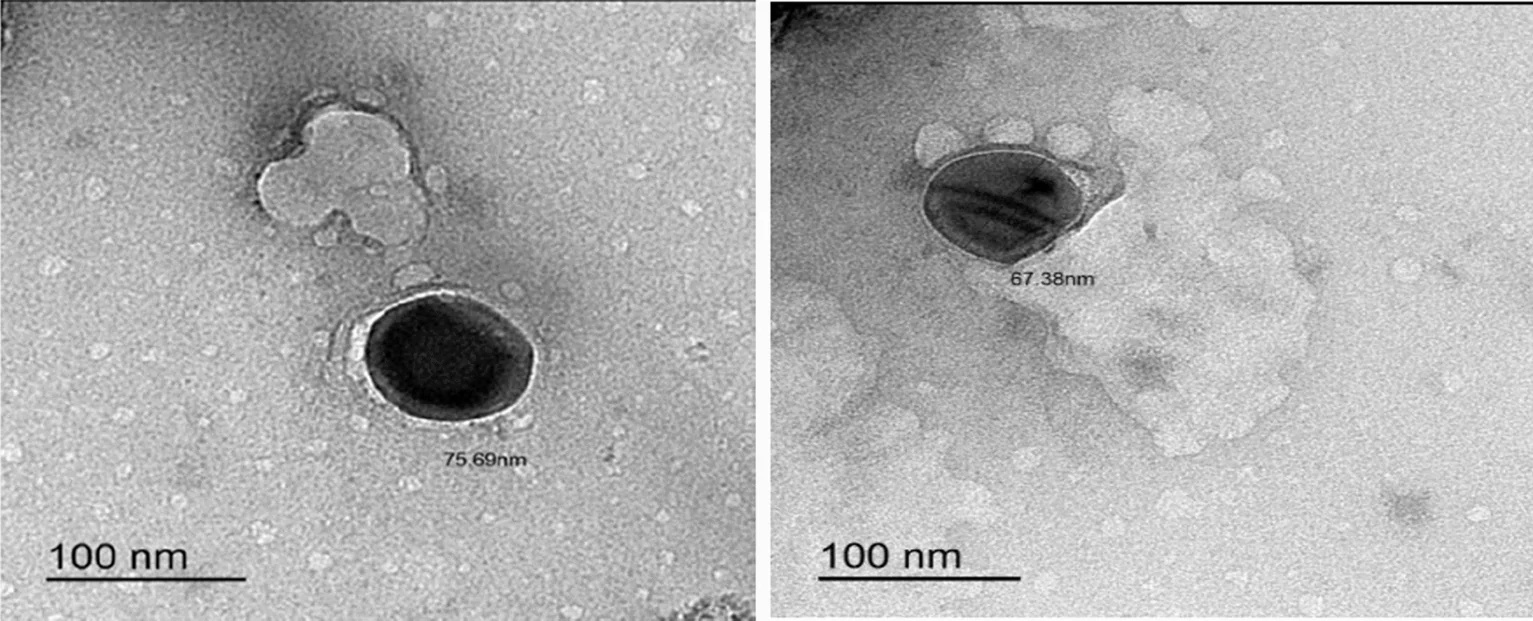
TEM (Transmission electron microscopy) of garlic volatile oil nanoemulsion.

Figure 8 present TEM micrographs illustrating the morphology and structural features of garlic essential oil nanoemulsion droplets. The images reveal that the droplets are predominantly spherical and are uniformly dispersed throughout the emulsion matrix.

The observed particle sizes, which ranged from 67.38 nm to 75.69 nm, indicate a narrow size distribution at the nanoscale.

###### TEM (transmission electron microscopy) of lemon volatile oil nanoemulsion

**Fig. 9:**
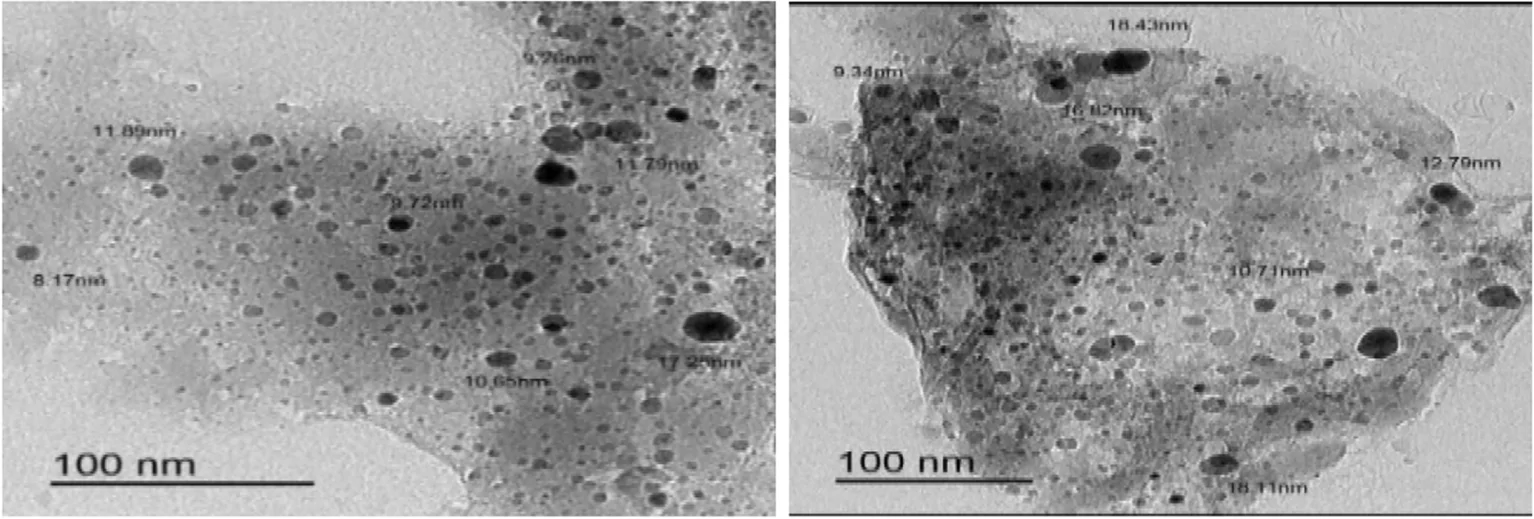
Transmission electron microscopy (TEM) of lemon essential oil nanoemulsion.

TEM analysis revealed that the lemon essential oil nanoemulsion was characterized by a uniformly structured system of nanosized droplets with a distinct spherical morphology evenly distributed throughout the emulsion. The particle sizes varied between 7.76 nm and 18.90 nm, as depicted in Fig. 9. These findings confirm the nanoemulsion’s uniform structure and efficient formation at the nanoscale, providing reassurance about the system’s stability.

##### Zeta potential

The zeta potential is a method for measuring the surface charge of particles in a liquid.

Zeta potential is used to predict dispersion stability, and its value depends on the physicochemical properties of the drug, polymer, and vehicle, as well as the presence of electrolytes and their adsorption. The Malvern Zeta sizer instrument measures it.

###### Zeta potential garlic essential oil nanoemulsion

**Fig. 10:**
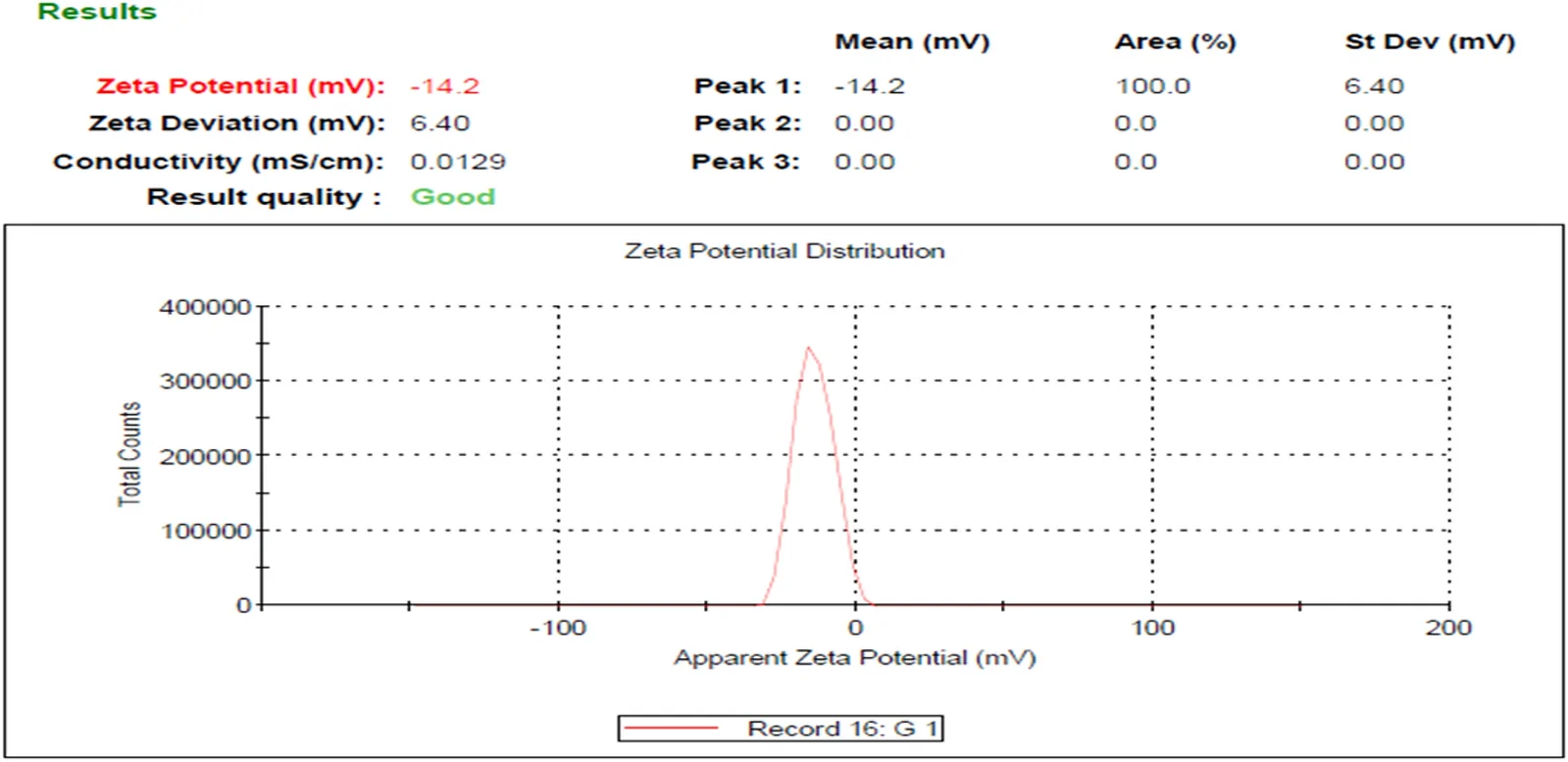
Zeta potential of garlic essential oil nanoemulsion at zero time.

**Fig. 11:**
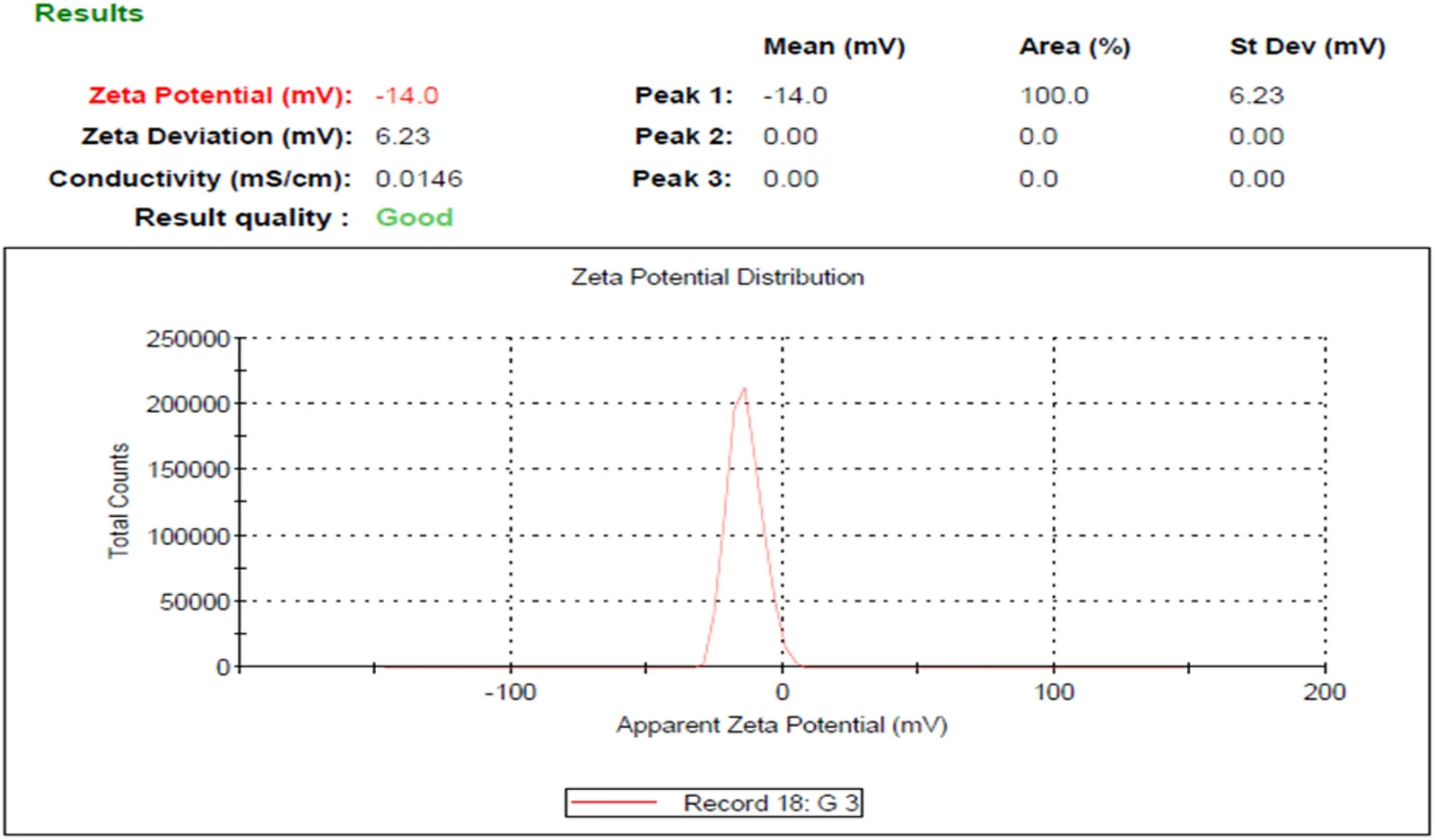
Zeta potential of garlic essential oil nanoemulsion at zero time.

**Fig. 12:**
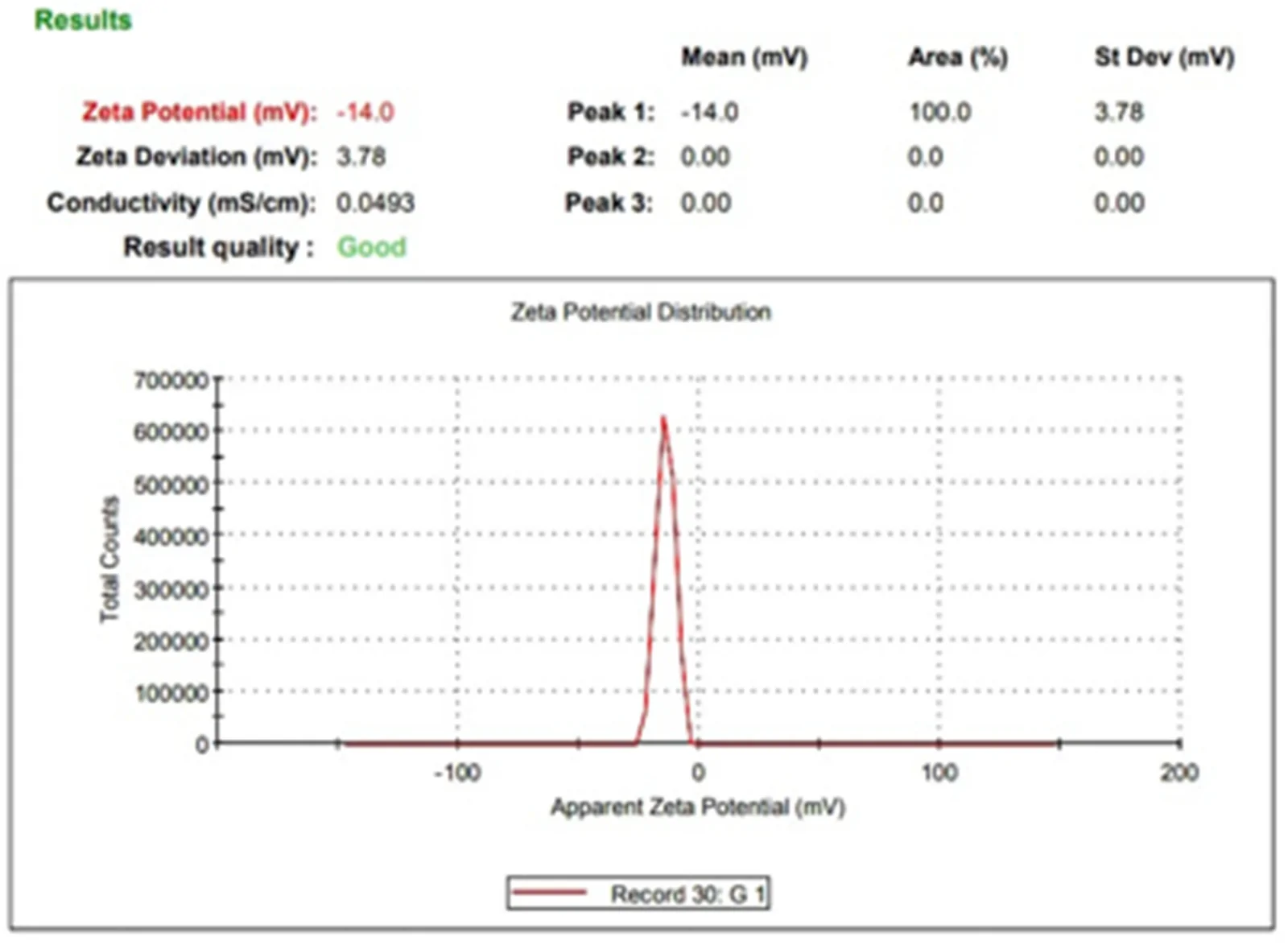
Zeta potential of garlic essential oil nanoemulsion at zero time.

At zero time, all three results of zeta potential for garlic essential oil nanoemulsion displayed negative zeta potential, with values of zeta potential -14.2 mV, -13.0 mV, and -14.0 mV (average -13.7 mV), as shown in Figs. 10, 11, 12.

**Fig. 13:**
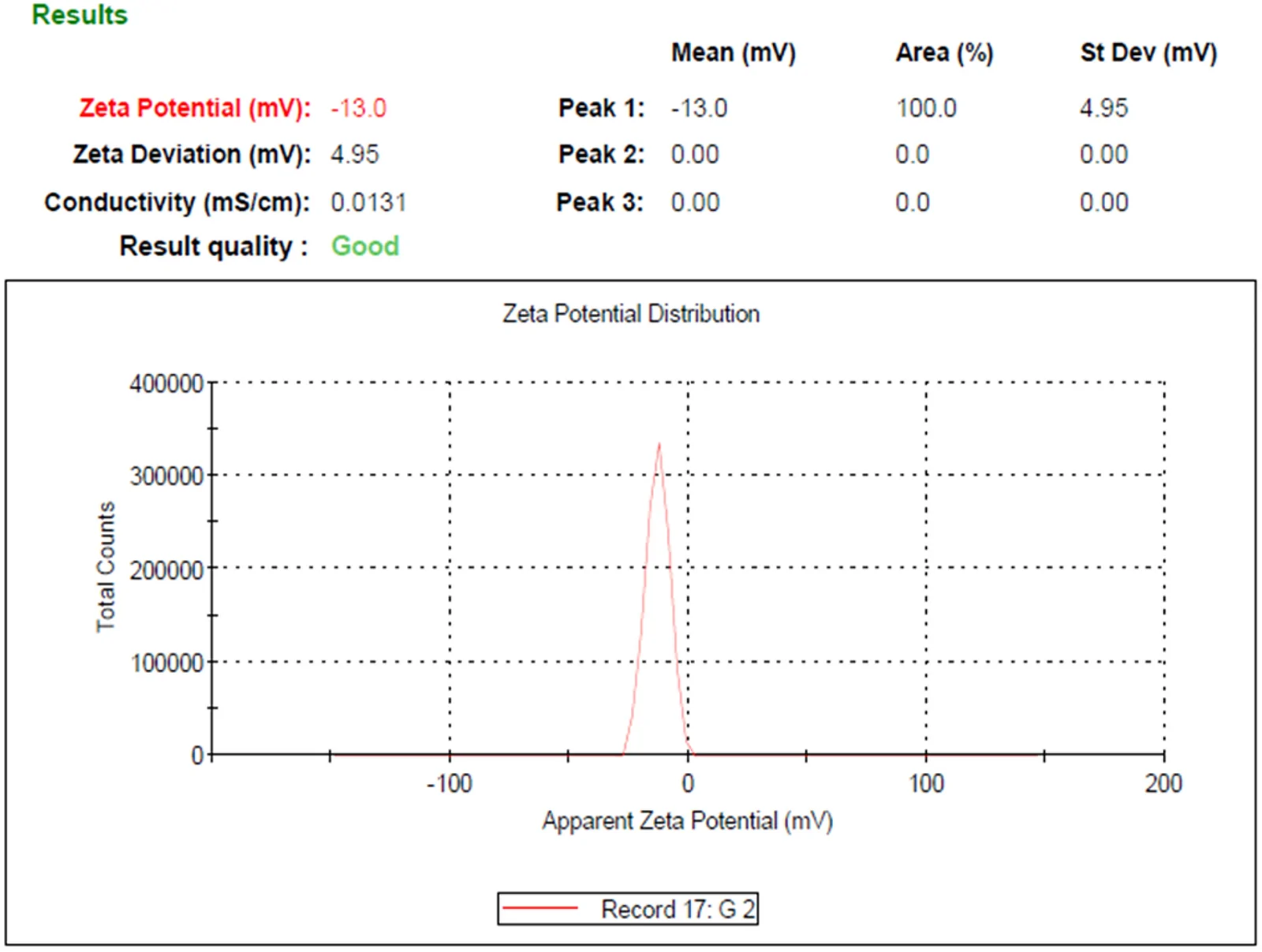
Zeta potential of garlic essential oil nanoemulsion at zero time.

**Fig. 14:**
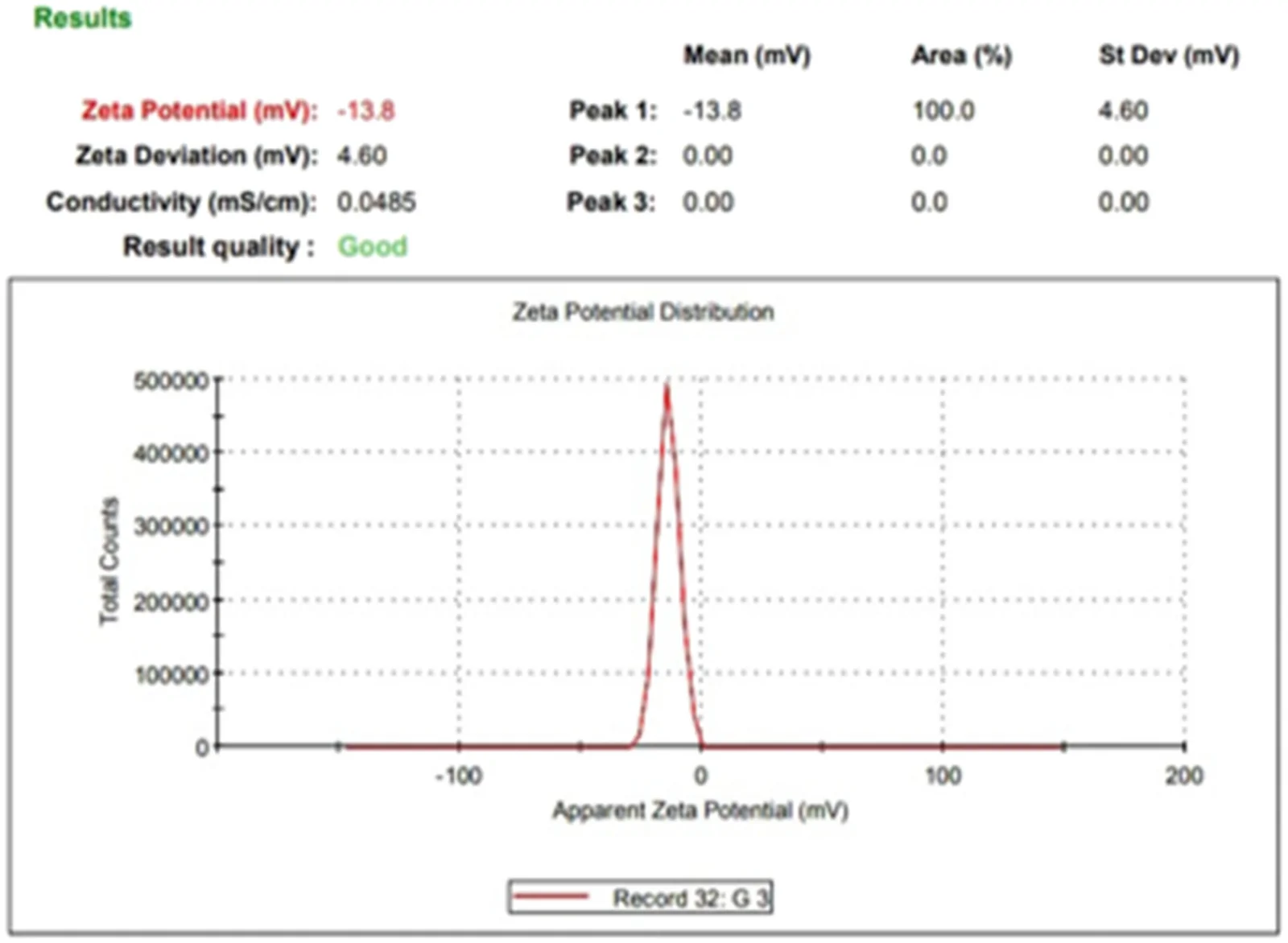
Zeta potential of garlic essential oil nanoemulsion after three months.

**Fig. 15:**
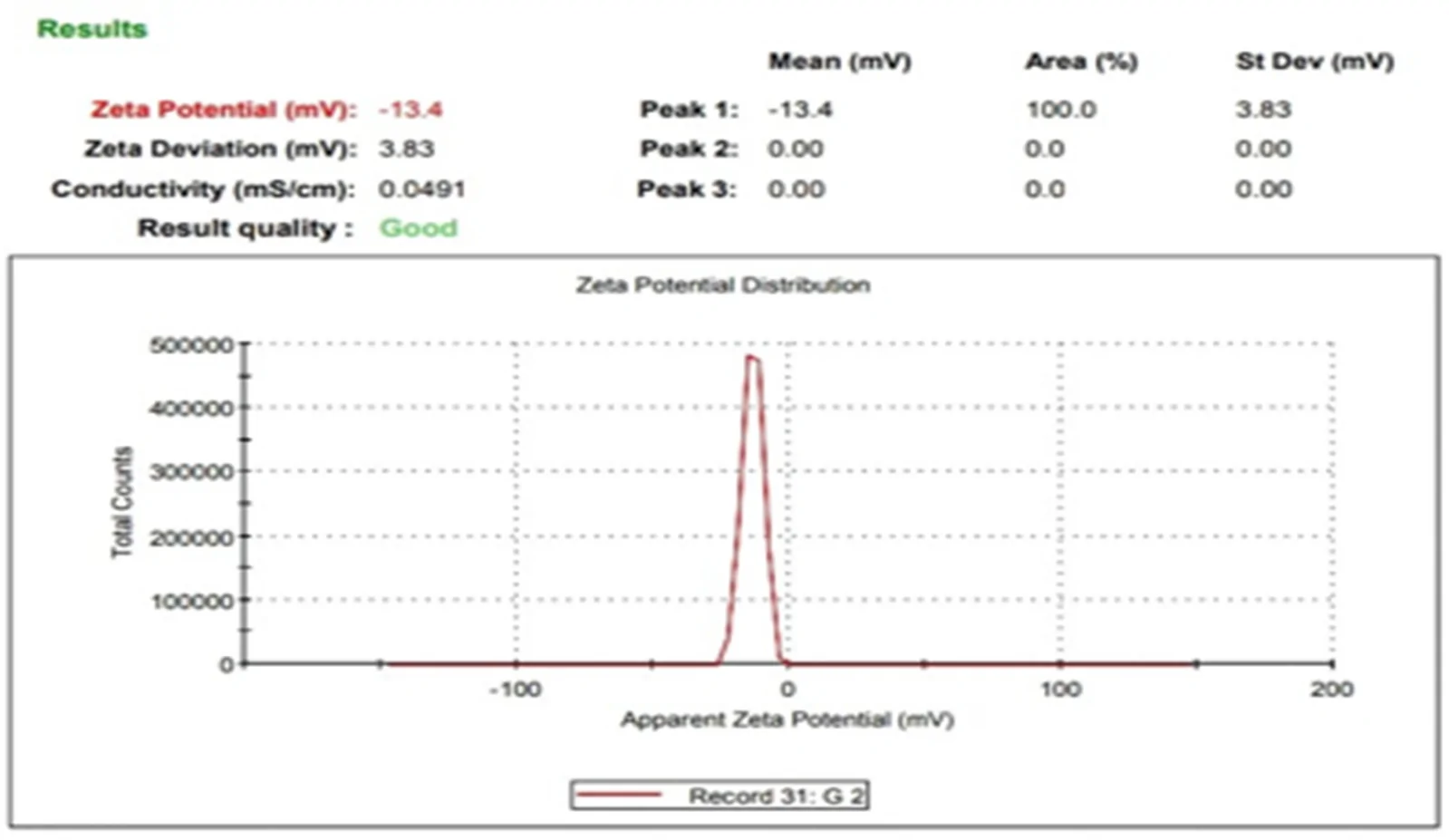
Zeta potential of garlic essential oil nanoemulsion after three months.

After three months, the zeta potential of garlic essential oil nanoemulsion was re-evaluated, and it was performed three times; all three results of zeta potential for garlic essential oil nanoemulsion displayed negative zeta potential, with values of zeta potential -14.0 mV, -13.4 mV, and -13.8 mV (average -13.7 mV), as shown in Figs. 13, 14, 15.

###### Zeta potential of lemon essential oil nanoemulsion

**Fig. 16:**
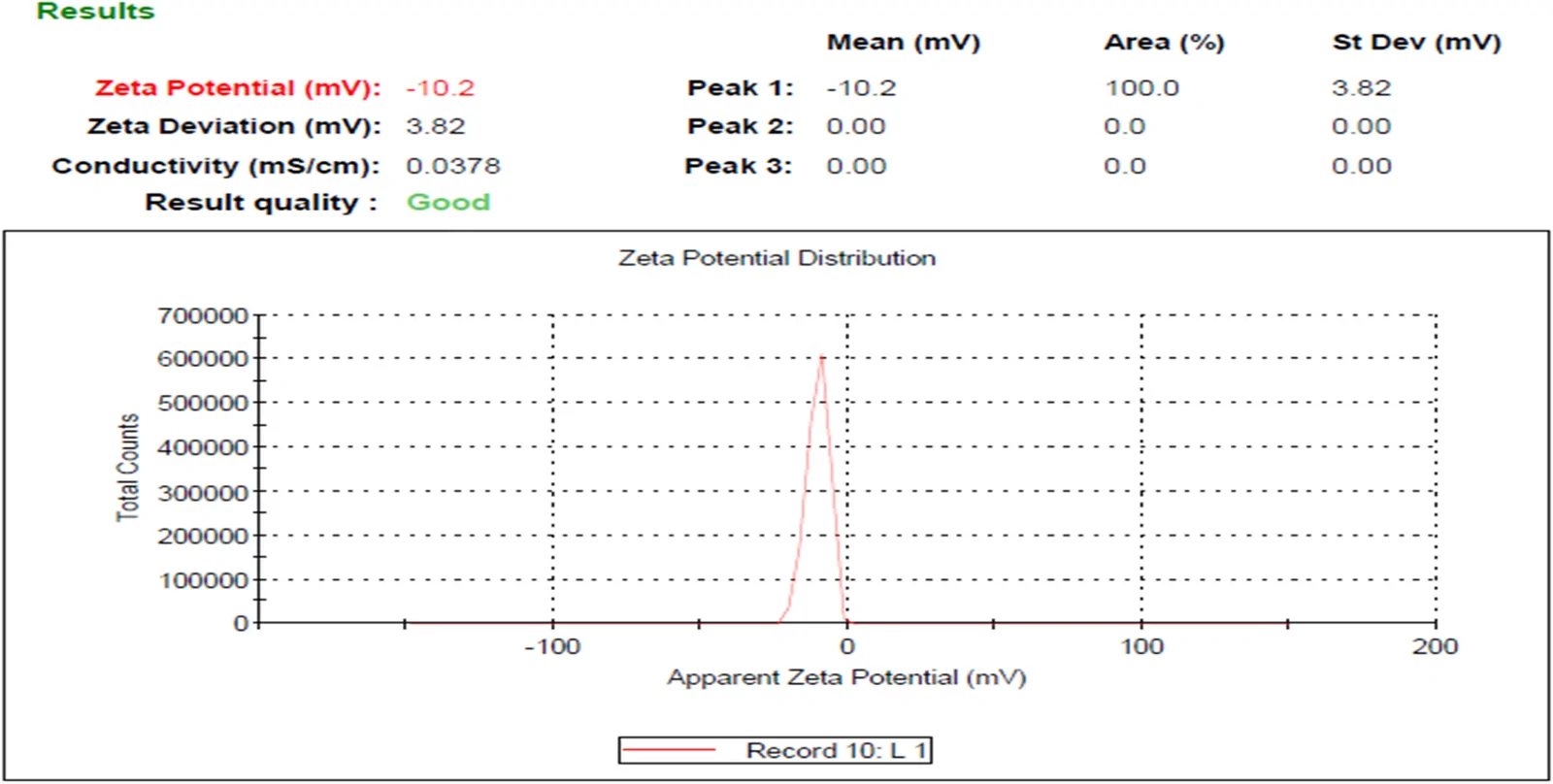
Zeta potential of garlic essential oil nanoemulsion after three months.

**Fig. 17:**
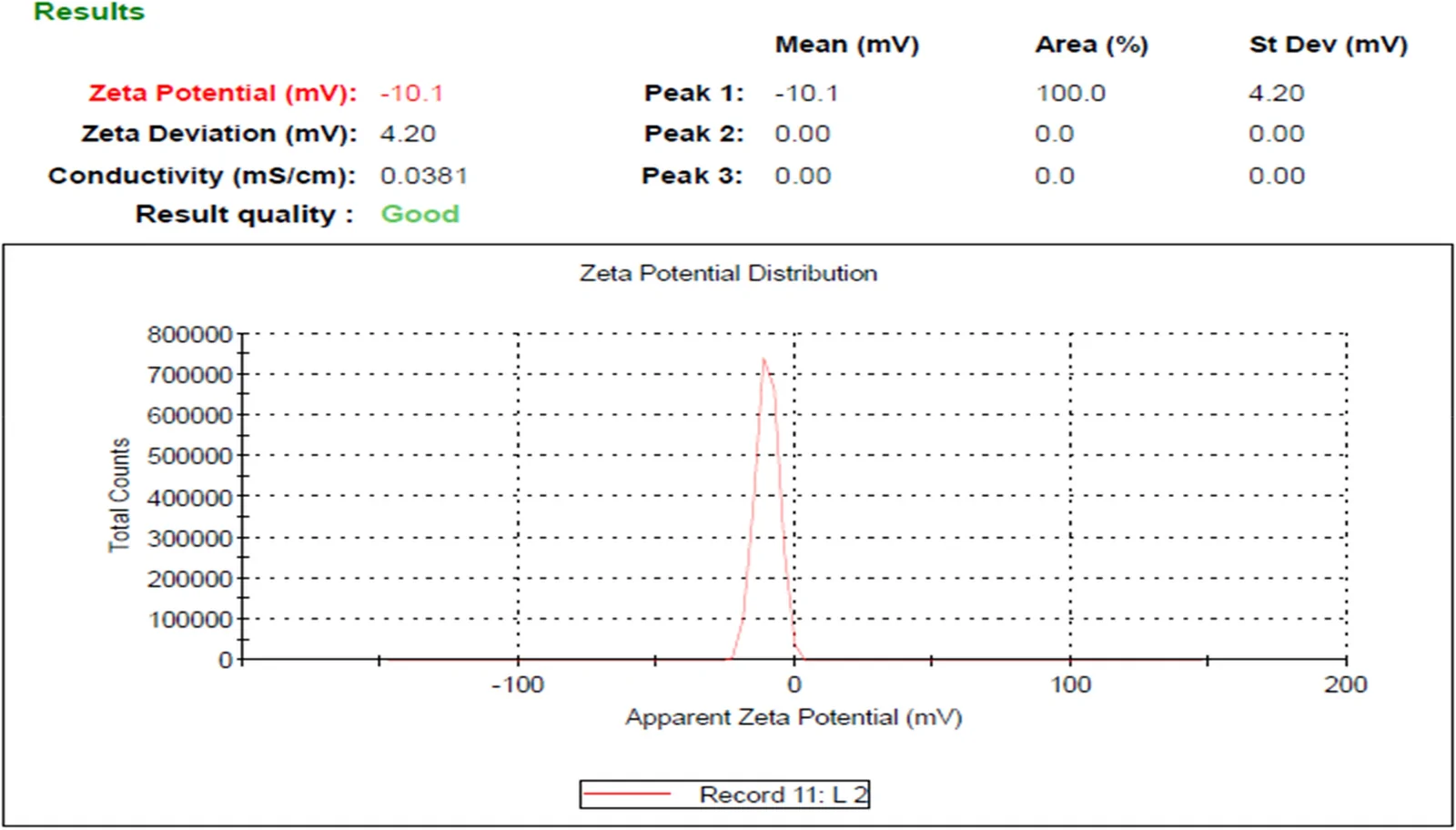
Zeta potential of lemon essential oil nanoemulsion.

**Fig. 18:**
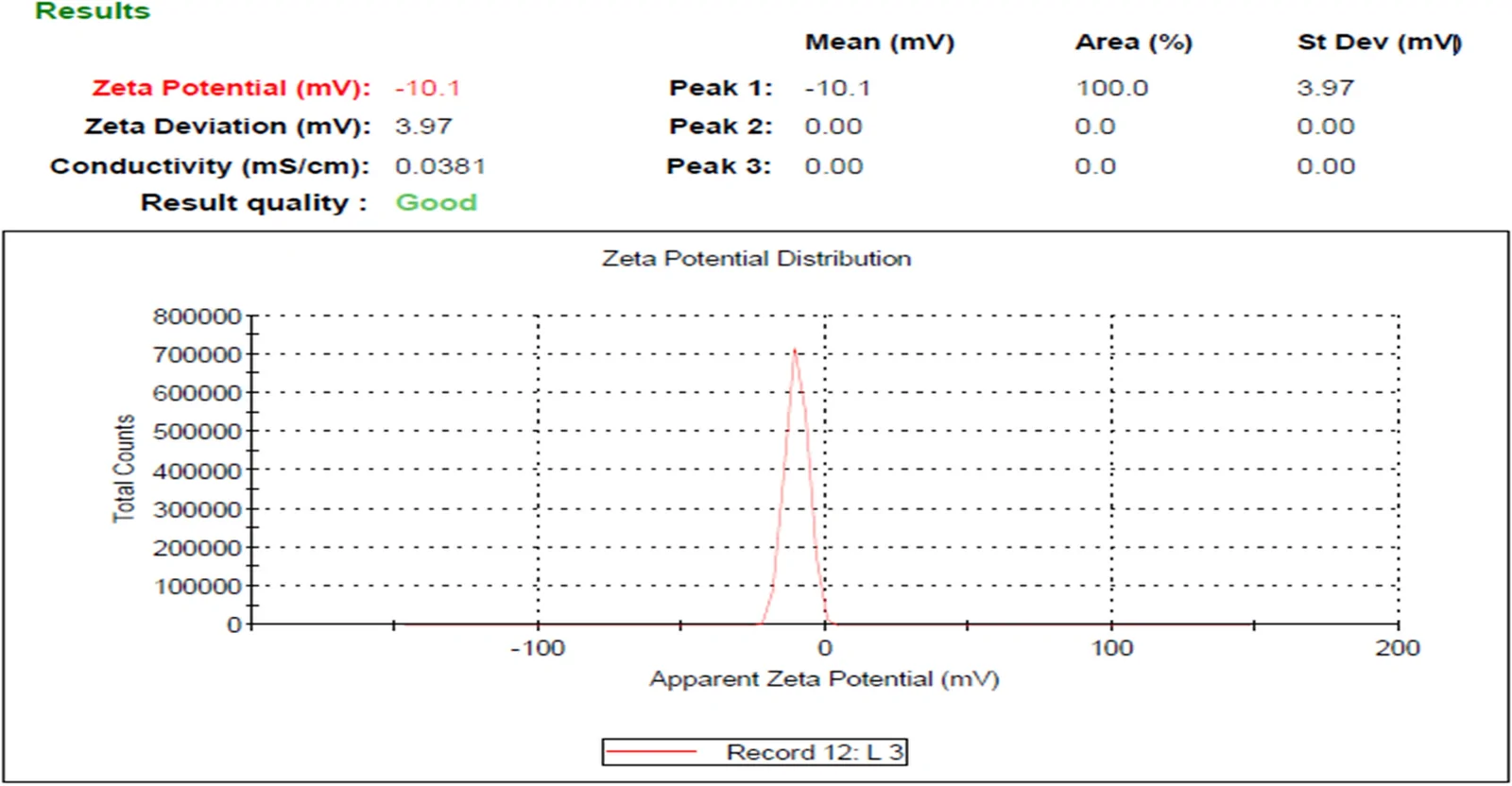
Zeta potential of lemon essential oil nanoemulsion.

Zeta potential for lemon essential oil nanoemulsion is shown in Figs. 16, 17, 18; three experiments were performed at 25 °C after dilution (100-fold); all three results displayed negative zeta potential, with zeta potential values of -10.2 mV, -10.1 mV, and -10.1 mV.

#### Chemical properties of regular and nano-volatile oils

The chemical properties of the oils were determined using established standard methods, as outlined for each respective parameter. The parameters analyzed included acid value (free fatty acids) and peroxide value.

##### Chemical properties of normal and nano garlic and lemon volatile oils

**Fig. 19:**
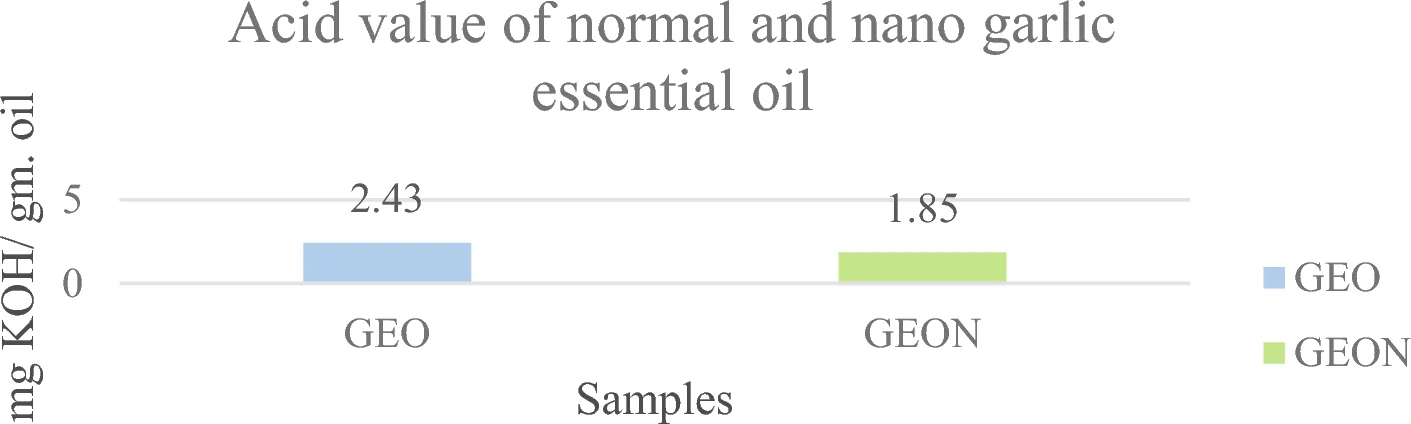
Zeta potential of lemon essential oil nanoemulsion.

**Fig. 20:**
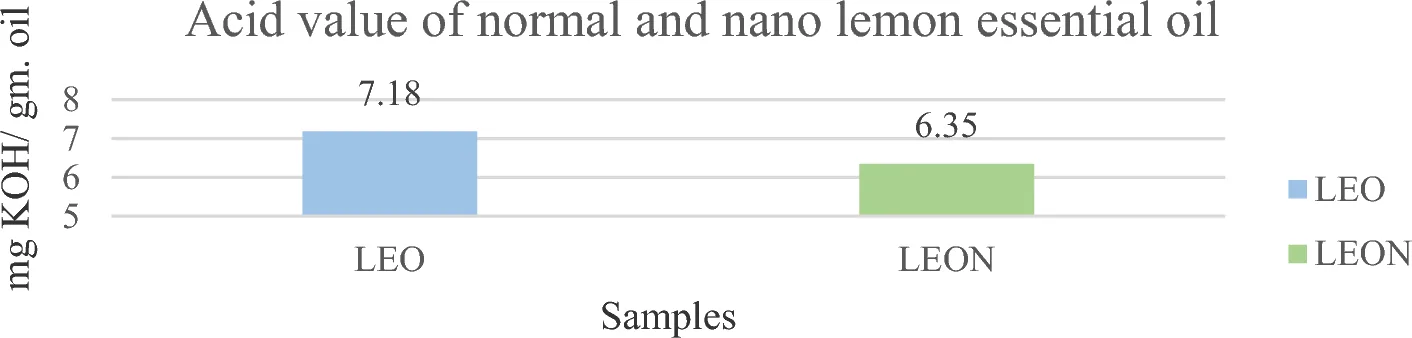
Acid value of normal and nano essential oils (garlic and lemon).

**Fig. 21:**
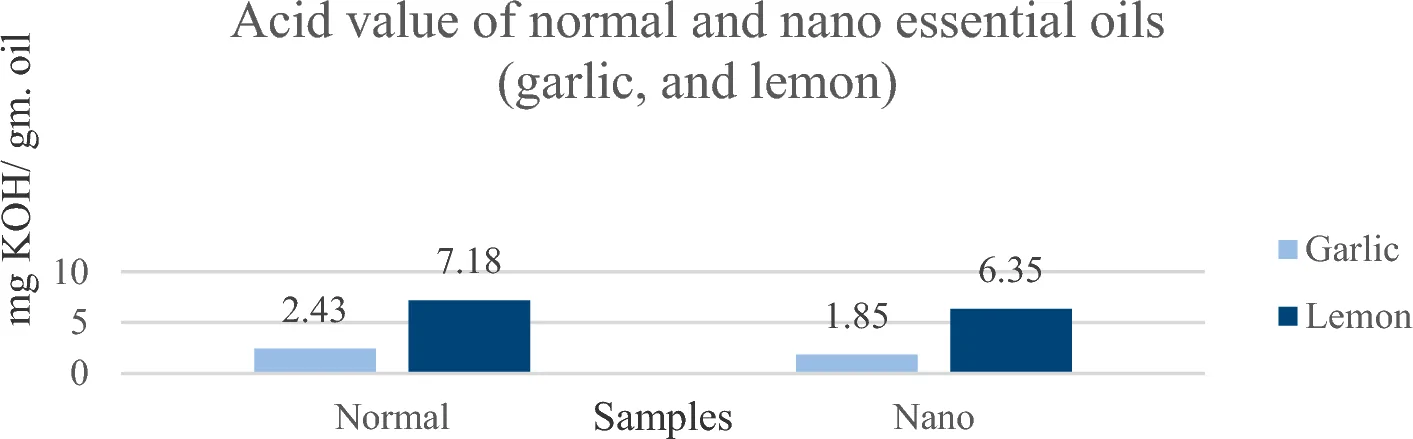
Peroxide value of normal and nano garlic essential oil. (GEO): garlic essential oil (GEON): garlic essential oil nanoemulsion.

**Fig. 22:**
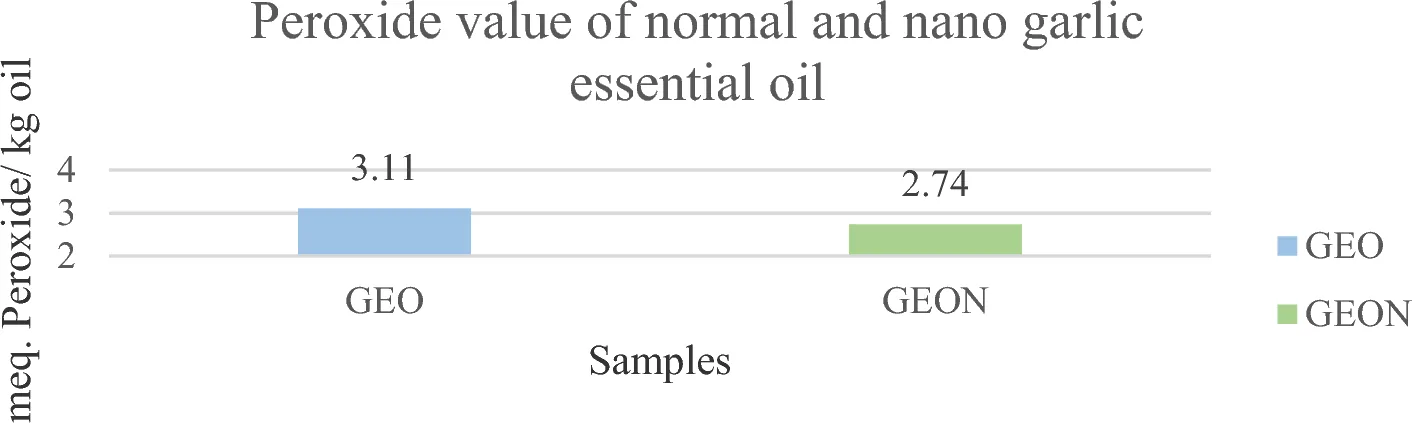
Peroxide value of normal and nano lemon essential oil (LEO): lemon essential oil (LEON): lemon essential oil nanoemulsion.

**Fig. 23:**
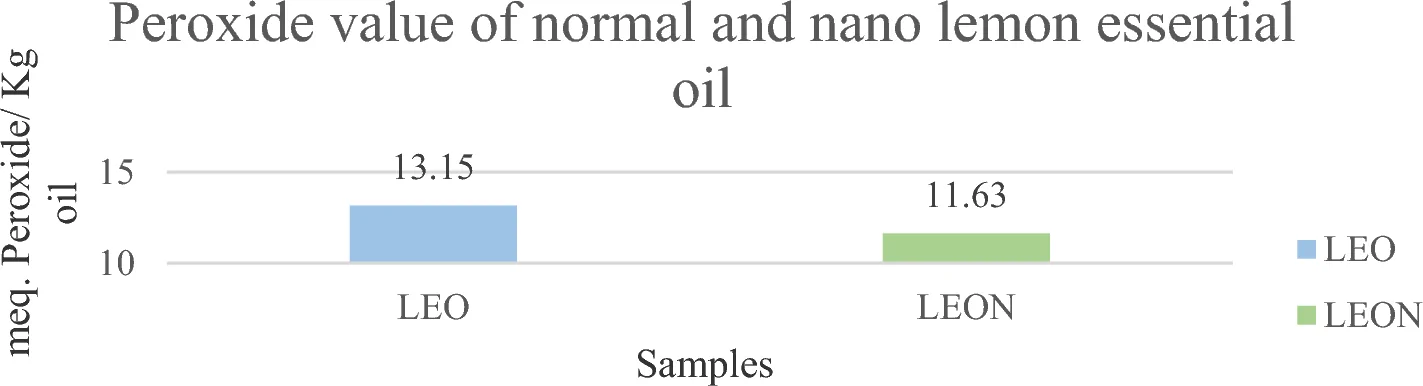
Peroxide value of normal and nano essential oils (garlic and lemon).

**Fig. 24:**
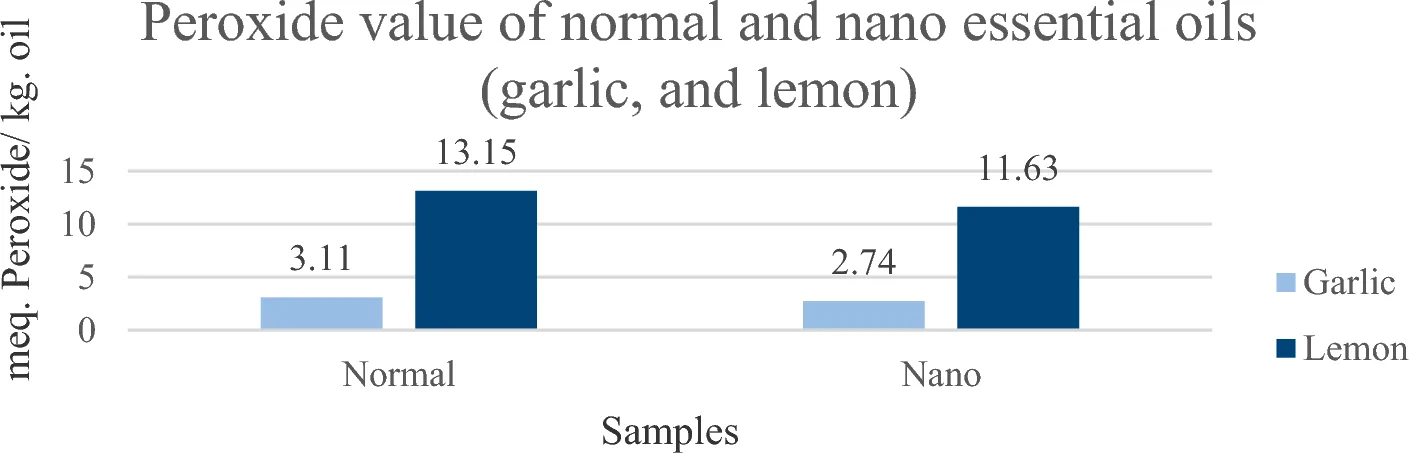
DPPH of regular and nano garlic essential oil. (GEO): garlic essential oil (GEON): garlic essential oil nanoemulsion.

The chemical properties of garlic essential oil (acid value and peroxide value) are presented in Tables 3 and 4 and Figs. 19, 20, 21, 22, 23, 24.

**Table 4:** Peroxide value of regular and nano volatile oils.

Oils	Normal	Nano	LSD _at 5%_
Lemon essential oil	13.15^a^ ± 0.060	11.63^b^ ± 0.101	0.10
Garlic essential oil	3.11^a^ ± 0.050	2.74^b^ ± 0.055	0.01

Significant values are different letters.

###### Acid value

Table 3 and Figs. 19 and 21 provide the acid values for normal and nano garlic essential oils.

The acid value of normal garlic essential oil (GEO) was 2.43 ± 0.085 mg KOH/g oil, whereas that of the garlic essential oil nanoemulsion (GEON) was 1.85 ± 0.091 mg KOH/g oil.

Table 3 and Figs. 20 and 21 provide the acid values for normal and nano lemon essential oils. The acid value of normal lemon essential oil (LEO) was 7.18 ± 0.065 mg KOH/g, while the nanoemulsion (LEON) exhibited a reduced acid value of 6.35 ± 0.060 mg KOH/g.

###### Peroxide value

The peroxide values for both garlic essential oil (GEO) and its nanoemulsion (GEON) are summarised in Table 4 and illustrated in Figs. 22 and 24. The results reveal that the peroxide value of garlic essential oil (GEO) was 3.11 ± 0.050 meq peroxide/kg oil, whereas the peroxide value for the garlic essential oil nanoemulsion (GEON) was 2.74 ± 0.055 meq peroxide/kg oil.

The peroxide values for normal and nano lemon essential oils are summarised in Table 4 and illustrated in Figs. 23 and 24. Regular lemon essential oil (LEO) showed a peroxide value of 13.15 ± 0.060 meq. Peroxide/kg oil, whereas the peroxide value of the lemon essential oil nanoemulsion (LEON) was significantly lower at 11.63 ± 0.101 meq. Peroxide/kg oil.

#### Free radical scavenging assay (DPPH) of normal and nano essential oils

**Fig. 25:**
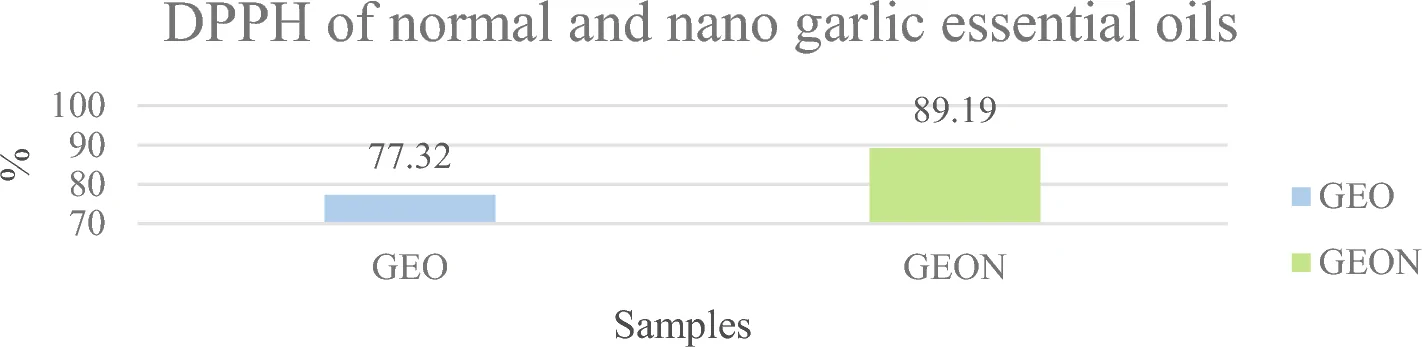
DPPH of regular and nano lemon essential oil. (LEO): lemon essential oil (LEON): lemon essential oil nanoemulsion.

**Fig. 26:**
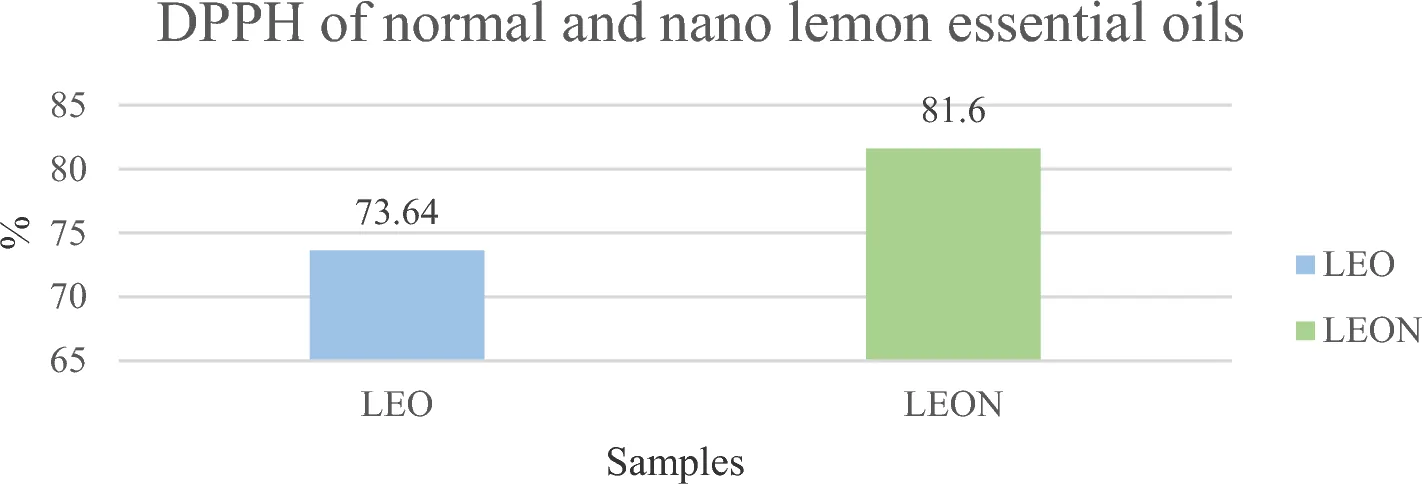
Free radical scavenging assay (DPPH) of regular and nano essential oils.

**Fig. 27:**
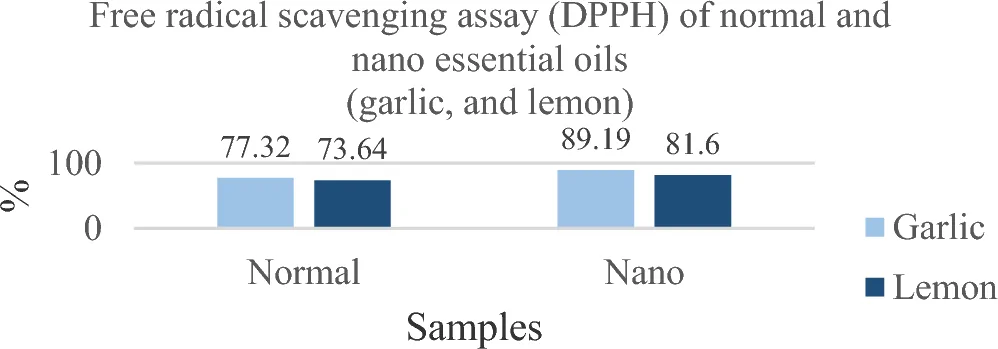
Free radical scavenging assay (DPPH) of regular and nano essential oils.

The DPPH assay is a well-established and reliable method for assessing the antioxidant capacity of bioactive compounds. It measures a substance’s ability to neutralize free radicals, highly reactive molecules with unpaired electrons, contributing to oxidative stress and cellular damage. The data are represented in Table 5 and Figs. 25, 26, and 27.

**Table 5:** Free radical scavenging assay (DPPH) of regular and nano volatile oils.

Oils	Normal	Nano	LSD _at 5%_
Lemon essential oil	73.64^b^ ± 0.870	81.60^a^ ± 1.104	4.89
Garlic essential oil	77.32^b^ ± 0.919	89.19^a^ ± 0.531	1.02

Significant values are different letters.

##### DPPH of regular and nano garlic essential oil

Table 5 and Figs. 25 and 27 illustrate the free radical scavenging activity of both regular and nanoemulsified garlic essential oils (GEO and GEON), as assessed by the DPPH assay. The data reveal that GEO exhibited a DPPH inhibition percentage of 77.32% ± 0.919, while GEON demonstrated a significantly higher inhibition of 89.19% ± 0.531.

##### DPPH of regular and nano lemon volatile oil

Table 5 and Figs. 26 and 27 illustrate the antioxidant capacity of both regular and nano lemon essential oils (LEO and LEON) as measured by the DPPH assay. The results revealed that the DPPH radical scavenging activity of regular lemon essential oil (LEO) was 73.64% ± 0.870, whereas the nanoemulsion form (LEON) exhibited a higher activity of 81.60% ± 1.104.

All results are presented as means ± SD (n = 3), and different superscript letters (a, b, c, …) indicate significant change at p˂0.05.

#### Biological effect of normal and nano essential oils on breast cancer cells (MCF-7)

The MCF-7 cell line is well-known as a dependable in vitro system for investigating breast cancer and assessing potential anticancer compounds.

##### Biological effect of normal and nano garlic essential oil on breast cancer cells

**Fig. 28:**
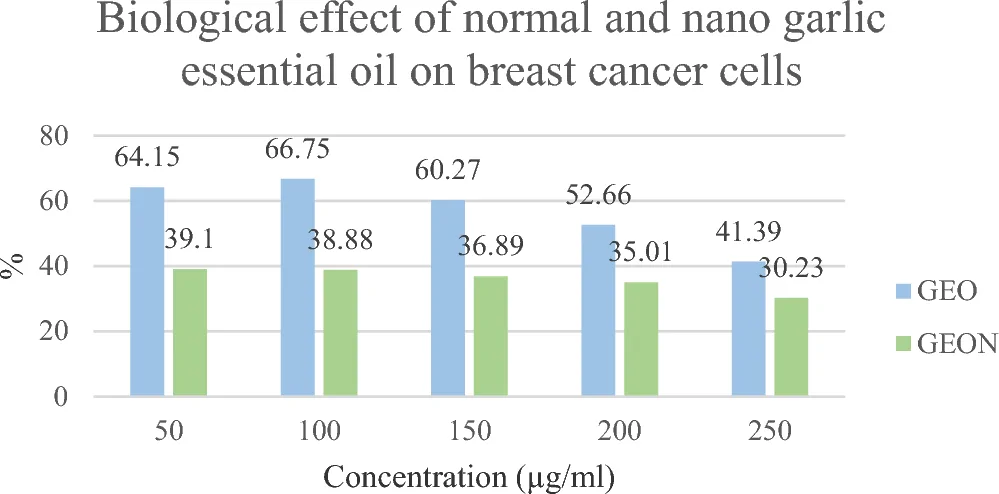
Biological effect of normal and nano garlic essential oil on breast cancer cells. (GEO): garlic essential oil (GEON): garlic essential oil nanoemulsion.

**Fig. 29:**
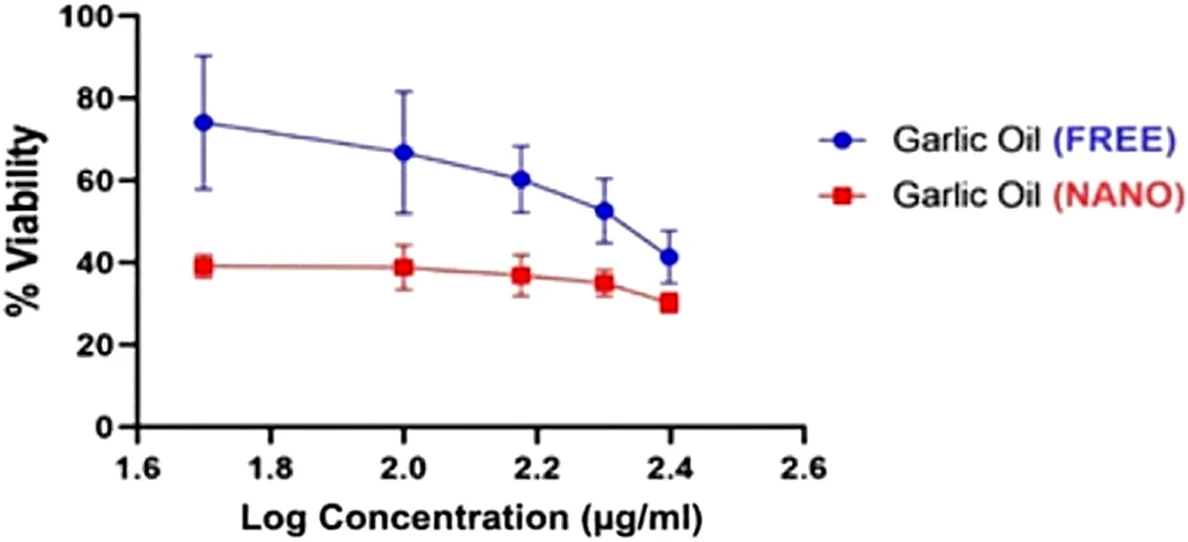
Biological effect of normal and nano garlic essential oil on breast cancer cells.

Table 6 and Figs. 28 and 29 present the effects of normal and nano garlic essential oil on the MCF-7 breast cancer cell line. The study tested both GEO and GEON at five concentrations: 50, 100, 150, 200, and 250 μg/ml.

**Table 6:** Biological effect of normal and nano garlic essential oil on breast cancer cells.

Concentration (µg/ml)	GEO	GEON	LSD _at 5%_
50	74.15 ^a^ ± 1.010	39.10 ^b^ ± 2.736	9.22
100	66.75 ^a^ ± 14.903	38.88 ^a^ ± 5.386	7.5
150	60.27 ^a^ ± 8.063	36.89 ^b^ ± 4.995	9.52
200	52.66 ^a^ ± 7.860	35.01 ^b^ ± 3.198	11.92
250	41.39 ^a^ ± 6.442	30.23 ^a^ ± 2.319	5.11

Values are expressed as mean ± SD, n = 3. Means with different letters are significantly different at p ≤ 0.05. (GEO): garlic essential oil (GEON): garlic essential oil nanoemulsion.

The percentage of viable breast cancer cells (MCF-7) at concentrations (50, 100, 150, 200, and 250 µg/mL) of regular garlic essential oil (GEO) was (74.15, 66.75, 60.27, 52.66, and 41.39%), respectively. The viability percentages at identical concentrations (50, 100, 150, 200, and 250 µg/mL) of garlic essential oil nanoemulsion (GEON) were recorded as (39.10, 38.88, 36.89, 35.01, and 30.23%), respectively. Concentrations (50, 150, and 200 μg/mL) of normal and nano garlic essential oil (GEO and GEON) demonstrated a significant difference in cell viability percentages; at concentrations (100 and 250 μg/mL), no significant difference was observed in cell viability percentages between normal and nano garlic essential oil (GEO and GEON).

**Fig. 30:**
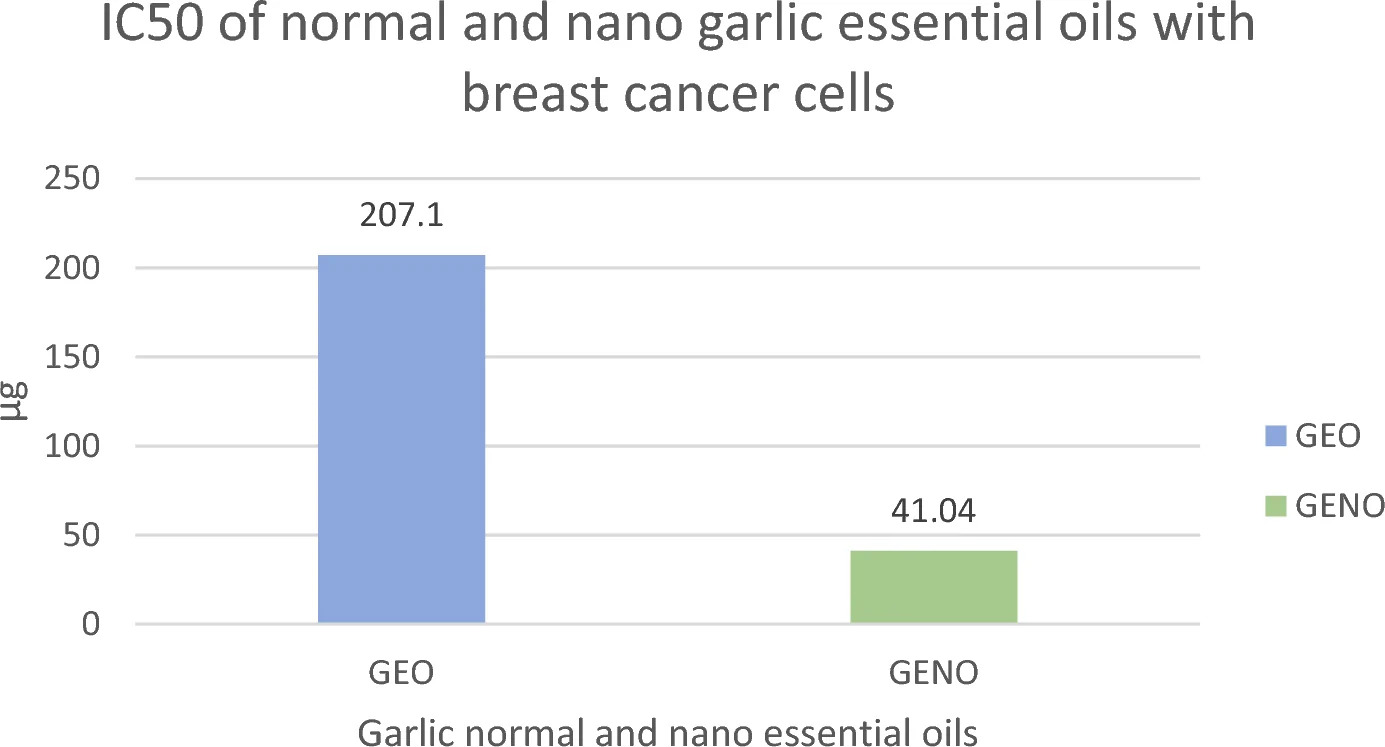
IC_50_ of normal and nano garlic essential oils with breast cancer cells. (GEO: garlic essential oil (GENO): garlic essential oil nanoemulsion.

Figure 30 showed the amount needed of normal and nano garlic essential oil (GEO and GEON) to destroy 50% of breast cancer cells (MCF-7); this is called IC_50._ (207.1 μg/mL) was the IC50 of normal garlic essential oil (GO). In contrast, the IC50 of garlic essential oil nanoemulsion was 41.04 μg/mL.

Table 8 and Figs. 34, 35, 36 show the results of IC_50_ to garlic oil compared to the results of lemon oil.

##### Biological effect of normal and nano lemon essential oil on breast cancer cells

**Fig. 31:**
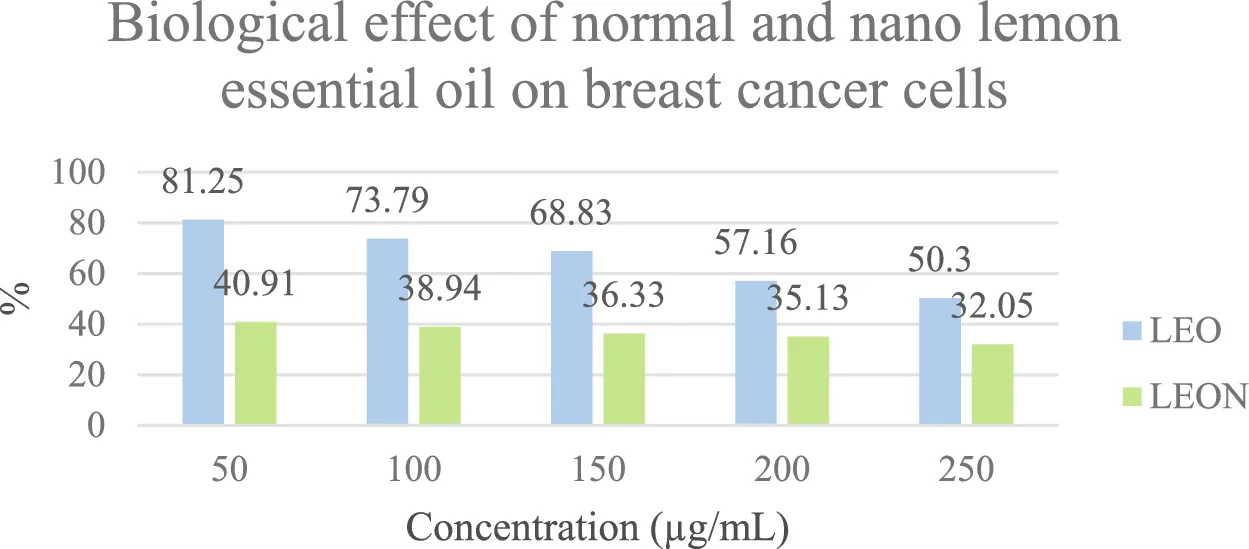
Biological effect of normal and nano lemon essential oil on breast cancer cells. (LEO): lemon essential oil (LEON): lemon essential oil nanoemulsion.

**Fig. 32:**
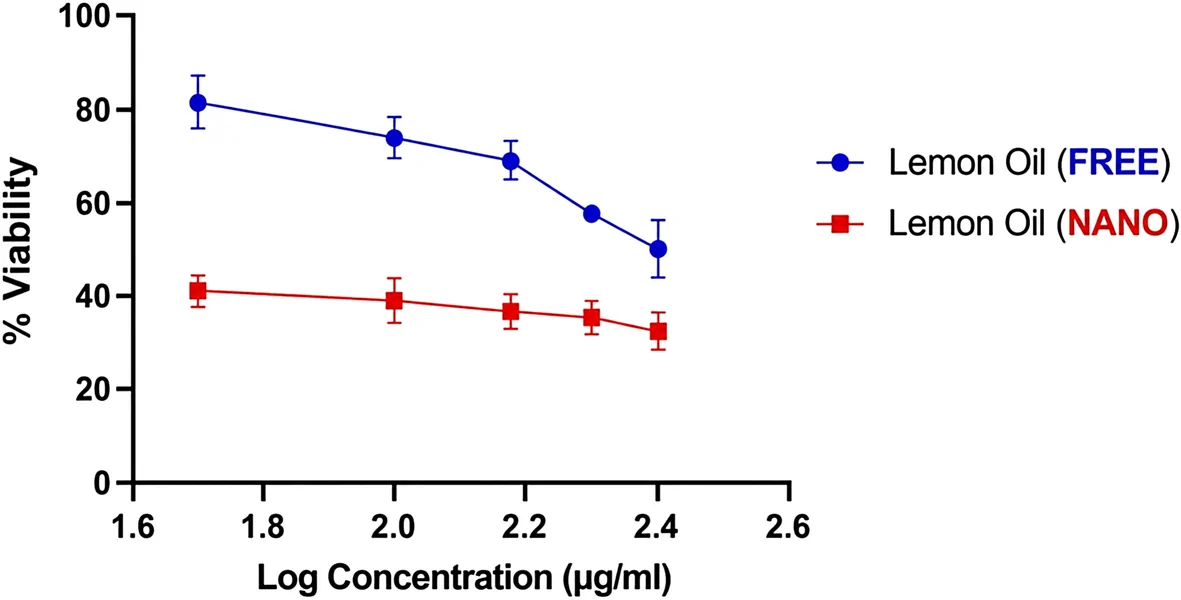
Biological effect of normal and nano lemon essential oil on breast cancer cells.

Table 7 and Figs. 31 and 32 show the result of the effect of normal and nano lemon essential oil (LEO and LEON) on breast cancer cell lines (MCF-7). This was determined at 5 concentrations (50,100,150, 200, and 250 μg/mL) of normal and nano lemon essential oil.

**Table 7:** Biological effect of normal and nano lemon essential oil on breast cancer cells.

Concentration (µg/ml)	LEO	LEON	LSD _at 5%_
50	81.25 ^a^ ± 5.412	40.91 ^b^ ± 2.319	10.53
100	73.79 ^a^ ± 4.152	38.94 ^b^ ± 4.870	9.11
150	68.83 ^a^ ± 3.085	36.33 ^b^ ± 3.062	5.10
200	57.16 ^a^ ± 1.910	35.13 ^b^ ± 2.381	6.24
250	50.30 ^a^ ± 6.759	32.05 ^b^ ± 3.019	12.51

Values are expressed as mean ± SD, n = 3. Means with different letters are significantly different at p ≤ 0.05. (LEO): lemon essential oil (LEON): lemon essential oil nanoemulsion.

The viability percentage of breast cancer cells (MCF-7) at concentrations (50, 100, 150, 200 and 250 µg/mL) of normal lemon essential oil (LEO) was (81.25, 73.79, 68.83, 57.16, 50.30%) respectively, while the viability percentage of breast cancer cells was (40.91, 38.94, 36.33, 35.13 and 32.05%) respectively, at same concentrations of lemon essential oil nanoemulsion (LEON) (50, 100, 150, 200 and 250 µg/mL).

There was a significant difference in cell viability percentage between normal and nano lemon essential oil (LEO and LEON), and between all concentrations (50, 100, 150, 200, and 250 μg/mL).

**Fig. 33:**
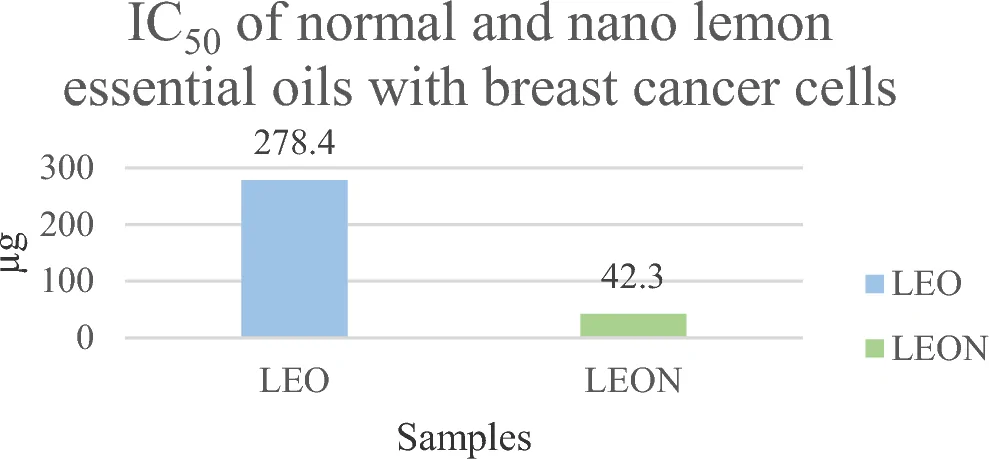
IC_50_ of normal and nano lemon essential oils with breast cancer cells. (LEO): lemon essential oil (LEON): lemon essential oil nanoemulsion.

Figure 33 showed the values of IC_50_ of normal and nano lemon essential oil (LEO and LEON), which were (278.4 μg/mL) for normal lemon essential oil (LEO), and (42.3 μg/mL) for lemon essential oil nanoemulsion (LEON).

The nanoemulsified formulation demonstrated a significantly lower IC_50_ value compared to its conventional counterpart, indicating enhanced cytotoxic potency.

Also, that means lemon essential oil nanoemulsion (LEON) is more effective on breast cancer cells than normal lemon essential oil (LEO). This may be due to the effect of nanoparticle size.

**Fig. 34:**
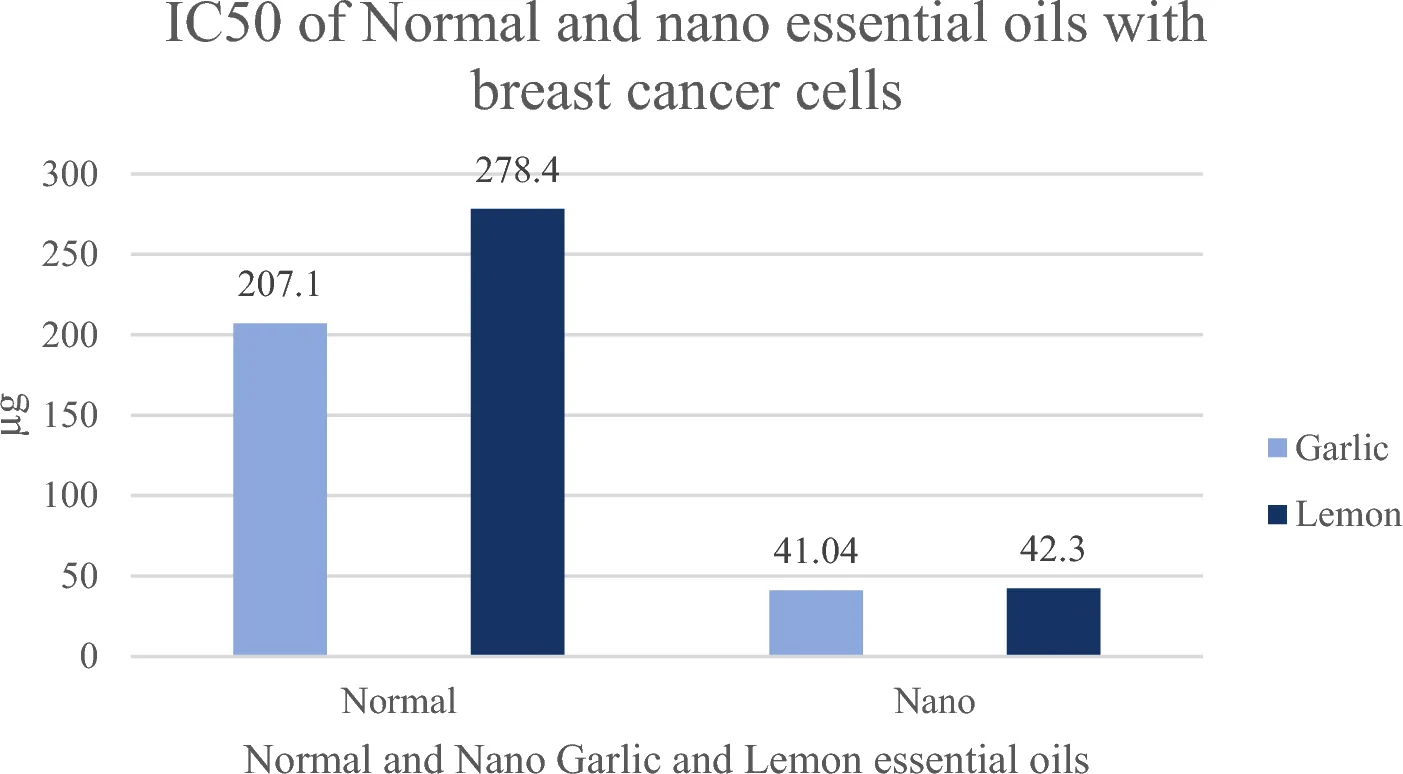
IC_50_ of normal and nano essential oils with breast cancer cells.

**Fig. 35:**
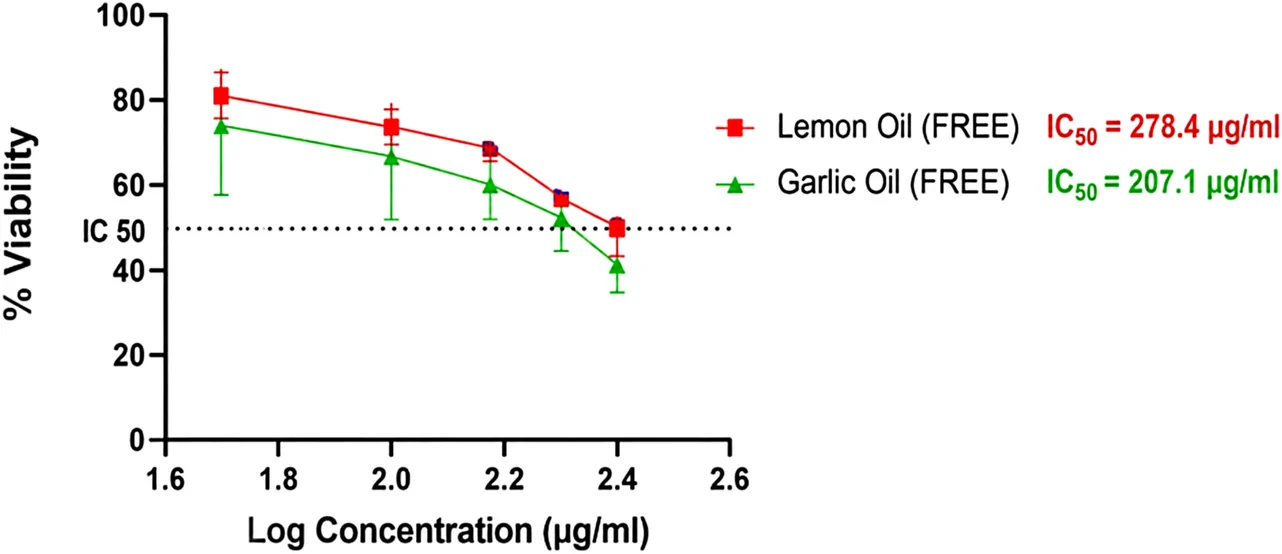
IC_50_ of normal essential oils with breast cancer cells.

**Fig. 36:**
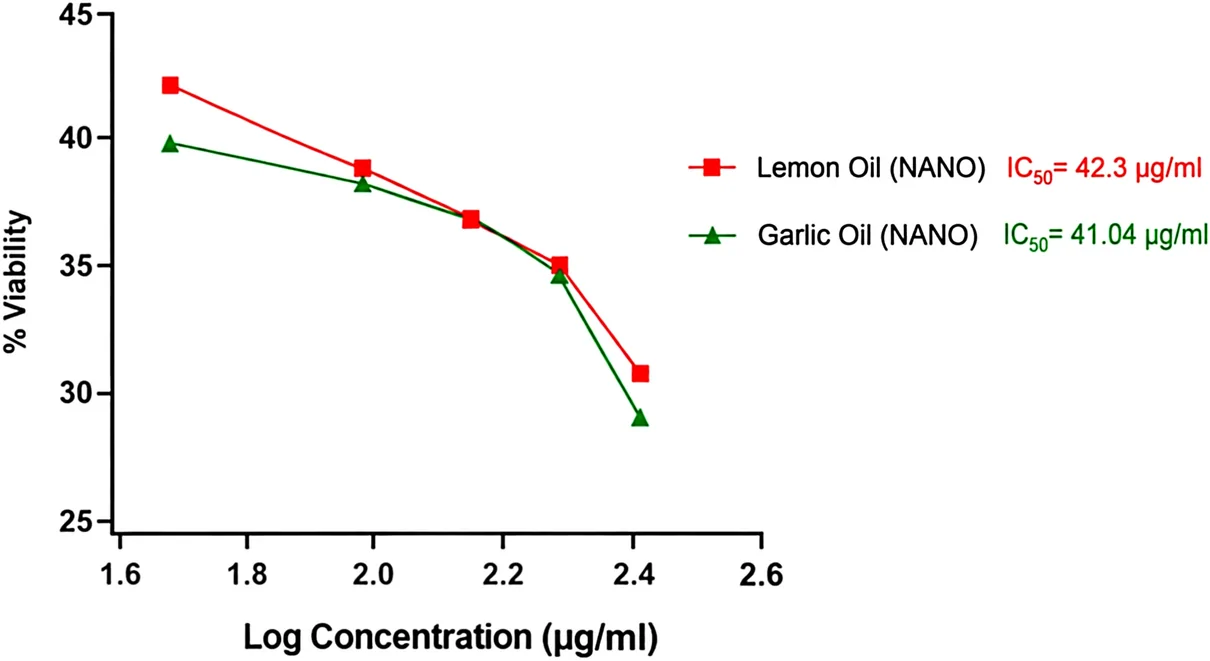
IC_50_ of nano-essential oils with breast cancer cells.

Table 8 and Figs. 34, 35, 36 show the results of IC_50_ for lemon essential oil compared to the results of garlic essential oil.

**Table 8:** IC_50_ of normal and nano essential oils with breast cancer cells.

Oils (µg/mL)	Normal	Nano
Garlic essential oil	207.1 ^a^	41.04 ^b^
Lemon essential oil	278.4 ^a^	42.3 ^b^

Significant values are different letters.

## Comparison of IC_50_ of normal and nano garlic and lemon essential oils with breast cancer cells

### Discussion

#### Fractionation and characterization of essential oil compounds using GC/MS

GC/MS is instrumental in analyzing terpenes, fatty acids, esters, aldehydes, and alcohols. The concentrations of these compounds can vary due to several factors, such as seasonal changes, metabolic adaptations, the specific plant parts being used, and the methods of extraction or distillation, along with other environmental or processing conditions^18^.

##### Identification and characterization of chemical components in garlic essential oil

Table 1 and Figs. 1 and 2 illustrate that in the GC/MS analysis of garlic essential oil (GEO), significant peaks were observed at retention times of 17.659 and 23.497 min, representing two primary compounds with relative abundances of 22.54% and 22.68%, respectively. The first compound, identified as methyl 2-propenyl trisulfide (C₄H₈S₃, molecular weight 152.30 g/mol), was followed by di-2-propenyl trisulfide (C₆H₁₀S₃, molecular weight 178.30 g/mol) as the second compound. These results confirm that both sulfur-containing compounds are the principal constituents of garlic essential oil.

The mass spectrum for methyl 2-propenyl trisulfide, the first major component, reveals several significant peaks: 152 (M)^+^, 105 (M-SCH_3_)^+^, 87 (M-S_2_H)^+^, 73 (M-CS_2_H_3_)^+^, and 64 (S_2_)^+^, with the base peak at 87 m/z depicted in Fig. 3. For di-2-propenyl trisulfide, the fragmentation pattern includes peaks at 178 (M)^+^, 137 (M–C₃H₅)^+^, 113 (M–S₂H)^+^, and 73 (C₃H₅S)^+^, where the most intense signal (base peak) occurs at 113 m/z, as shown in Fig. 4. Table 1 provides comprehensive details on the names, chemical formulas, and molecular weights of these two key components^19–21^.

The variations in concentrations of garlic essential oil components between this study and prior research highlight the impacts of geographical and environmental factors on stability and quality assessments. Notably, the composition of garlic bulbs is significantly influenced by soil, weather, and climate conditions. Garlic contains a wide variety of bioactive sulfur compounds, such as diallyl sulfide (DAS), allicin (AC), S-allyl cysteine (SAC), alliin, diallyl disulfide (DADS), and diallyl trisulfide (DATS)^19^.

Findings from this research correlate with previous studies stating that the primary components of garlic essential oil include trisulfide, methyl 2-propenyl (29.12%), trisulfide, di-2-propenyl (21.98%), and diallyl disulfide (17.24%)^20^***.*** While garlic oils from different regions exhibit similar qualities, the variation in organosulfur compound quantities likely influences their therapeutic and sensory properties^21^***.***

The transformation of alliin to allicin occurs when the enzyme alliinase is released upon crushing the garlic bulb. Due to its instability, allicin rapidly converts into sulfur derivative components like allin. A significant mechanism for the efficacy of sulfur compounds is the donation of a hydrogen atom from a reactive thiol (-SH) to a free radical, thereby neutralizing the reactive radical and forming a less reactive thiyl radical. This process halts the free radical chain reaction, which otherwise could lead to damage in biological molecules such as lipids and DNA^22,23^.

##### Identification and characterization of chemical components in lemon volatile oil

The findings from the GC/MS analysis of lemon essential oil are presented in Table 2 and Figs. 5, 6 and 7). In lemon essential oil, significant peaks were observed at a retention time of 13.057 min, corresponding to compounds that exhibited a relative abundance of 56.09%. This particular compound has a molecular formula of C₁₀H₁₆ and a molecular weight of 136.23 g/mol, confirming that D-limonene is the primary component identified in the oil.

The fragmentations of D-limonene reveal important peaks at 136 (M)^+^, 121 (M-CH₃)^+^, 93 (M-C₃H₇)^+^, and 77 (C₆H₅)^+^, with the base peak observed at 93 m/z, as displayed in Fig. 7. 

These results align with previous research, highlighting that D-limonene is indeed the main component of lemon peel essential oil found in Marrakech, with a concentration of 39.49%^24^. Furthermore, the chemical composition of lemon essential oil can vary significantly due to several factors, including geographical location, climate, harvest time, plant age, and distillation method. This observation supports the findings of the current study^25^.

#### Characterization of volatile oils nano-emulsion

##### TEM (transmission electron microscopy)

Transmission electron microscopy (TEM) is a precise technique that involves directing a focused beam of high-energy electrons through an ultrathin specimen mounted on a copper grid. This interaction between the electron beam and the atomic structure of the sample allows for the creation of high-resolution images at the nanoscale. While TEM shares similarities with conventional light microscopy, its reliance on electrons instead of light allows for superior magnification and resolution^26^.

###### TEM of garlic volatile oil nanoemulsion

Figure 8 illustrate the characteristics of a garlic essential oil nanoemulsion, demonstrating a narrow size distribution. This indicates the successful formation of a stable nanoemulsion composed of well-defined, spherical nanoparticles. According to Zhang et al., garlic essential oil nanoemulsions formulated with Tween 80 (a non-ionic surfactant) showcase exceptional stability, maintaining homogeneous droplets with no significant changes observed over a storage period of up to 30 days^27^.

###### TEM of lemon volatile oil nanoemulsion

Figures 9 depict the TEM analysis of lemon essential oil nanoemulsions (LEO-NEs), confirming that the droplets are predominantly spherical and uniformly distributed. However, the particle sizes varied, ranging from 7.76 nm to 18.90 nm. This finding is consistent with the results reported by Sultan et al.^28^.

The analysis suggests that the nanoemulsions of both garlic and lemon essential oils fall within the nanoscale range and exhibit spherical morphology. While the lemon essential oil nanoemulsion displayed the smallest particle size, the garlic essential oil nanoemulsion had a relatively larger size. These observations align with previous studies, reinforcing the validity of the current research.

##### Zeta potential

To measure zeta potential, the nanoemulsion is diluted, and its value is estimated from the electrophoretic mobility of oil droplets. A zeta potential of ± 30 mV is generally considered sufficient for ensuring the physical stability of a nanoemulsion^29^.

###### Zeta potential of garlic essential oil nanoemulsion

The zeta potential for garlic essential oil nanoemulsion is illustrated in Figs. 10, 11, 12, 13, 14 and 15. The electrical charge of the nanoparticles is crucial for the stability of the nanoemulsion. The zeta potential was measured initially and after three months of storage at room temperature in a dry and dark location, with three replicates for each measurement.

The findings indicated that the zeta potential for garlic essential oil nanoemulsion remained consistent at both zero time and after three months of storage. This stability suggests that the nanoemulsion effectively maintained its properties during the storage period. The measurements were conducted at 25°C, and all samples underwent a 100-fold dilution. As established by Aguilar-Pérez et al.^30^, zeta potential values within ± 30 mV indicate good colloidal stability. Although the garlic nanoemulsion in this study exhibited a modest zeta potential of -13.7 mV—below the ± 30 mV threshold typically associated with good stability—it showed stability for three months^31,32^. This durability may be attributed to factors such as the presence of Tween 80, which enhances nanoemulsion stability by reducing interfacial tension and preventing droplet coalescence through steric hindrance^33^.

###### Zeta potential of lemon essential oil nanoemulsion

In contrast, the zeta potential for lemon essential oil nanoemulsion, shown in Figs. 16, 17, 18, revealed negative values across three separate experiments conducted at 25°C after a 100-fold dilution. The measured zeta potential values were -10.2 mV, -10.1 mV, and -10.1 mV. These results are consistent with previous reports, which indicated negative charges on the outer surface of lemon essential oil, particularly when combined with Tween 80 (− 2.30 mV)^34^.

In summary, all tested nanoemulsions (garlic and lemon) exhibited anionic charges within the ± 30 mV range. The garlic nanoemulsion demonstrated the highest charge and maintained good stability over three months, consistently displaying a zeta potential around -13.7 mV.

#### Chemical properties of regular and nano-volatile oils

This study specifically focuses on the acid value and peroxide value of oils. The **acid value** refers to the amount of potassium hydroxide (KOH) required to neutralize the free fatty acids (FFA) in one gram of oil or fat, serving as an important indicator of rancidity. Free fatty acids usually result from the hydrolysis of oil triglycerides^35^.

Moreover, peroxides are significant early products of autoxidation, with the peroxide value typically expressed in milliequivalents of oxygen per kilogram of fat^36^.

##### Chemical properties of normal and nano garlic and lemon volatile oils

The chemical properties, specifically the acid and peroxide values of lemon essential oil, are illustrated in Tables 3 and 4 and depicted in Figs. 19, 20, 21, 22, 23, 24.

###### Acid value

Data presented in Table 3 and Figs. 19 and 21 indicate a statistically significant difference in the acid values between regular and nano forms of garlic essential oil. Volatile oils with elevated acid values usually show lower storage stability. In line with this, garlic essential oil demonstrated good storage quality compared to oils high in unsaturated fatty acids, measuring a free fatty acid content of approximately 1.4 ± 0.05%, suggesting its suitability for consumption and potential for use as an edible oil in industrial applications^37^.

The findings correlate with previous research showing an acid value of 17.90 mg KOH/g for garlic essential oil, reinforcing its free fatty acid content profile. Higher acid values are commonly associated with reduced stability and a greater risk of rancidity. Garlic essential oil comprises volatile compounds and many free fatty acids. For an oil to be categorized as edible, its free fatty acid content should generally lie within the 1–3% range^38^.

The low acid value of garlic essential oil likely stems from significant bioactive compounds such as methyl 2-propenyl trisulfide, di-2-propenyl trisulfide, diallyl disulfide, and diallyl sulfide. Notably, the garlic essential oil nanoemulsion (GEON) displayed a lower level of rancidity compared to regular garlic essential oil (GEO), indicating enhanced quality over prolonged storage. This improved stability and lower rancidity can probably be attributed to nanotechnology, which optimizes the dispersion and protective qualities of the garlic oil particles^39^.

In addition, data from Table 3 and Figs. 20 and 21 reveal substantial differences that imply the nanoemulsion’s enhanced quality, likely due to nanotechnology’s stabilization effects. Moreover, the compound D-limonene, recognized for its bioactive properties, might also influence the acid value variations observed between regular and nanoemulsified forms.

In contrast, the findings for regular and nano lemon essential oils (LEO and LEON) show relatively high acid values, indicating a decline in quality, reduced edibility, and an increased risk of spoilage during storage. Oils are generally considered safe for consumption if their free fatty acid content is maintained within a 1–3% range. The heightened acid values of regular and nano lemon essential oils could result from the extraction method or extended storage conditions^38^.

The study’s results are bolstered by reports indicating an acid value of 8.68 KOH mg/g for lemon essential oil. The acid value serves as an essential parameter reflecting the concentration of free fatty acids, acting as a reliable indicator of oil quality. A lower acid value typically denotes better preservation and stability of the oil over time^40^.

###### Peroxide value

Determining the peroxide value (PV) provides valuable insight into the extent of primary oxidation in oil samples. The presence of double bonds in fats and oils makes them particularly susceptible to autoxidation, especially in oils with a higher degree of unsaturation. The peroxide value is a crucial measurement for assessing oxidative rancidity, as peroxides are key intermediates in this reaction^30^. Specifically, the peroxide value quantifies the levels of primary oxidation products, represented as milligrams of active oxygen present in one gram of the lipids^41^.

Analysis of Table 4 and Figs. 22 and 24 reveals statistically significant differences in the peroxide values of garlic essential oil (GEO) and its nanoemulsion (GEON), with the nanoemulsion displaying a lower peroxide value. This indicates reduced primary oxidation and suggests that the nanoemulsion formulation may enhance oxidative stability, contributing to better preservation and a longer shelf life compared to conventional garlic essential oil.

The peroxide value of garlic oil was measured at 247.12 ± 0.06, indicating a notably high value that signals the onset of oxidative rancidity. This reinforces the tendency of garlic oil to deteriorate rapidly when stored at room temperature. Typically, the peroxide values of fresh oils are expected to remain below 10 milliequivalents per kilogram. Values rising between 30 and 40 milliequivalents per kilogram result in oils developing a rancid flavor, which marks the onset of oxidation^42^. This observation aligns with trends identified in the current study.

Further examination of Table 4 and Figs. 23 and 24 highlights significant differences in the peroxide values of both lemon essential oil (LEO) and lemon essential oil nanoemulsion (LEON). The data suggest that the nanoemulsion form exhibits better oxidative stability. The reduced peroxide value in the nanoemulsion may be attributed to the stabilizing effects of nanotechnology on the oil, in conjunction with the antioxidant properties of D-limonene, the key active compound in lemon essential oil. Converting the oil into a nano-sized form likely enhances its oxidation resistance, thereby preserving its quality.

For reference, fresh oils typically exhibit peroxide values below 10 milliequivalents per kilogram, which indicates better freshness and stability^36^. The higher peroxide values noted in this study may be linked to factors such as the extraction method or prolonged storage, both of which can accelerate oxidation and compromise oil quality. The recorded peroxide value for lemon essential oil was 53.76 meq O2/kg, indicating a higher degree of oxidative degradation that may affect its overall freshness and suitability for use^40^.

Peroxide value serves as a crucial measure of primary oxidation in oils, providing early indications of rancidity, particularly in unsaturated fats and oils. The detection of peroxides marks the beginning of oxidative degradation. According to the International Olive Council (IOC) standards, essential oils with peroxide values exceeding 20 meq O2/kg are classified as less stable and possess significantly reduced shelf life. Limonene, a significant active compound found in citrus essential oils, is utilized in numerous products such as face washes, skincare oils, and insecticides due to its natural therapeutic and protective properties^40^.

D-Limonene, a monoterpene prevalent in the essential oils of citrus fruits, is celebrated for its antioxidant capabilities, particularly its effectiveness in inhibiting lipid peroxidation^43^. These properties align with the findings of the present study. Compared to pure lemon essential oils, lemon essential oil nanoemulsions demonstrate superior antioxidant properties. While pure lemon oil’s insolubility in aqueous solutions can limit its antioxidant effectiveness, the nanoemulsion form effectively addresses this limitation by ensuring excellent water solubility^44^**.** This improvement enables better delivery of bioactive compounds, with increased surface area facilitating more effective penetration of active ingredients, supporting the conclusions drawn in this research.

In summary, the chemical analysis of both regular and nanoemulsified essential oils (garlic and lemon) indicates that garlic essential oil (GEO) and its nanoemulsion (GEON) exhibit superior quality compared to lemon essential oil (LEO) and its nanoemulsion (LEON). This conclusion is supported by the significantly lower acid and peroxide values recorded for both forms of garlic essential oil, which remain well below the 3% threshold for freshness and stability, suggesting minimal signs of rancidity^38^**.**

#### Free radical scavenging assay (DPPH) of normal and nano essential oils

The DPPH assay measures a substance’s ability to neutralize free radicals, which are highly reactive molecules with unpaired electrons that contribute to oxidative stress and cellular damage. Antioxidants play a crucial role in mitigating these harmful effects by donating electrons or hydrogen atoms, stabilizing the free radicals and halting their propagation. The DPPH assay allows for a quantitative evaluation of the efficiency of antioxidants in scavenging free radicals, providing valuable insight into the protective potential of natural extracts and formulations^45^.

##### DPPH of regular and nano garlic essential oil

The free radical scavenging activity of conventional garlic essential oil (GEO) and its nanoemulsion (GEON), as determined by the DPPH assay, is summarized in Table 5 and illustrated in Figs. 25 and 27. Results showed a statistically significant difference between the two formulations, with GEON demonstrating superior scavenging activity compared to its conventional counterpart. This indicates that the process of nanoemulsification enhances the antioxidant performance of garlic essential oil.

Both GEO and GEON displayed considerable antioxidant capacity, primarily attributed to the high levels of organosulfur compounds, notably diallyl disulfide and diallyl trisulfide. These compounds are recognized as the key bioactive constituents contributing to the antioxidant properties of garlic essential oil^45^. They effectively neutralize reactive oxygen species (ROS) by donating electrons or hydrogen atoms, thereby reducing oxidative stress and safeguarding cellular macromolecules from oxidative damage. The findings align with previous research highlighting how garlic-derived organosulfur compounds contribute significantly to its antioxidant activity^39,46^

The enhanced antioxidant performance of GEON can likely be explained by the physicochemical advantages that come with nanoemulsification. The reduced droplet size increases the specific surface area, improves dispersion in the reaction medium, and enhances the accessibility and bioavailability of the antioxidant constituents, making interactions with free radicals more efficient. Moreover, studies suggest that nanoformulations can enhance the biological performance of garlic-derived bioactive compounds through improved stability, uniform distribution, and increased engagement with antioxidant pathways. These results corroborate earlier research demonstrating that garlic nanoparticles exhibit increased antioxidant activity compared to conventional formulations^47^.

##### DPPH of regular and nano lemon volatile oil

The DPPH scavenging activity of conventional lemon essential oil (LEO) and its nanoemulsion (LEON) is also outlined in Table 5 and depicted in Figs. 26 and 27. LEON exhibited significantly higher antioxidant activity than LEO, highlighting the effectiveness of nanoemulsification in enhancing the free radical scavenging capacity of lemon essential oil. This increase can be traced back to the reduced droplet size of the nanoemulsion. The smaller droplets increase the specific surface area, promote uniform dispersion, and improve the solubility and bioavailability of bioactive constituents.

The observed antioxidant activities in both LEO and LEON largely result from the high concentration of D-limonene, the predominant monoterpene present in lemon essential oil, known for its free radical scavenging properties^48^. Previous studies have documented that D-limonene exhibits concentration-dependent antioxidant activity in DPPH and other radical scavenging assays, where it neutralizes free radicals and inhibits oxidative chain reactions^49^. These findings reinforce the present results, confirming D-limonene’s pivotal role in contributing to the antioxidant potential of lemon essential oil.

Overall, the DPPH assay demonstrated that both garlic and lemon essential oils, in both their conventional and nanoemulsion forms, possess significant antioxidant activity owing to their characteristic bioactive constituents. Notably, nanoemulsification greatly enhanced the antioxidant performance of both oils. The superior activity of the nanoemulsions (GEON and LEON) can be attributed to their reduced particle size, which increases interfacial surface area and facilitates more effective interactions between antioxidant molecules and free radicals. Additionally, the improved physicochemical stability, uniform dispersion, and enhanced bioavailability of nanoemulsion systems are likely contributors to their superior radical-scavenging efficiency.

Among all formulations evaluated, garlic essential oil, especially in the form of its nanoemulsion (GEON), displayed the highest antioxidant activity, followed by conventional garlic essential oil (GEO). Conversely, lemon essential oil and its nanoemulsion (LEO and LEON) exhibited comparatively lower DPPH radical scavenging activity; however, nanoemulsification still significantly enhanced the antioxidant capacity of LEON relative to its conventional counterpart.

#### Biological effect of normal and nano essential oils on breast cancer cells (MCF-7)

Breast cancer remains the most frequently diagnosed malignancy among women worldwide, representing a significant public health challenge and highlighting the urgent need for more effective therapeutic strategies. The MCF-7 cell line, a human breast adenocarcinoma model, is extensively used for evaluating the anticancer potential of various compounds due to its reproducibility and clinical relevance. Chronic inflammation is known to play a critical role in tumor initiation, progression, and metastasis, making therapeutic agents with both anticancer and anti-inflammatory properties particularly appealing to researchers.

Natural products have historically been a valuable source for bioactive molecules in anticancer drug discovery. Recently, green nanotechnology, especially the creation of food-derived nanoformulations, has emerged as a promising approach to enhance the therapeutic efficacy of these natural compounds. This is attributed to their improved biocompatibility, low toxicity, environmental sustainability, and ability to increase the stability and bioavailability of bioactive constituents^50^

The cytotoxic activity of various formulations against MCF-7 cells was evaluated by determining the half-maximal inhibitory concentration (IC₅₀), which indicates the concentration needed to inhibit 50% of cell viability or proliferation compared to untreated control cells. The IC₅₀ value is a widely accepted quantitative measure of the antiproliferative potency of anticancer agents, where lower IC₅₀ values signify greater cytotoxic efficacy^51^.

##### Biological effect of normal and nano garlic essential oil on breast cancer cells

The data presented in Table 6 and Figs. 28 and 29 indicate that both normal garlic essential oil (GEO) and its nanoemulsion form (GEON) significantly reduce the viability of breast cancer cells, specifically the MCF-7 cell line. The study shows that increasing concentrations of both types of garlic oil lead to decreased cell viability, with GEON exhibiting a stronger effect than GEO.

At a concentration of 50 µg/mL, GEON resulted in a cell viability of 39.10%, compared to 41.39% at a higher concentration of 250 µg/mL for GEO. This suggests that even at lower concentrations, GEON can effectively target breast cancer cells, likely due to enhanced bioavailability and biological activity provided by the nanoemulsion.

In Table 8, the IC50 values further substantiate that GEON is more potent as an anticancer agent compared to GEO, requiring a smaller amount to achieve the same effect on cell viability. The study also compares the efficacy of garlic essential oil with lemon essential oil, although detailed results for the latter are mentioned in Table 8 and Figs. 34, 35, 36.

Moreover, when examining the cytotoxicity of *Allium sativum* L. extract on MCF-7 cells over different time frames (24, 48, and 72 h), a significant reduction in cell viability was observed, particularly at higher concentrations^52^. This aligns with previous studies suggesting that allicin and other sulfur compounds in garlic contribute to its anticancer properties by inhibiting mutagenesis and influencing cancer-related pathways^53,54^.

Overall, the study indicates that garlic essential oil, particularly in its nanoemulsified form, holds promise as a natural anticancer agent, thanks to its rich content of organosulfur compounds and a strong antioxidant profile^55^.

##### Biological effect of normal and nano lemon essential oil on breast cancer cells

In Table 7 and Figs. 31 and 32, the results indicate that increasing concentrations of both normal and nano lemon essential oil (LEO and LEON) lead to a decrease in the viability of breast cancer cells. Both forms demonstrate a positive effect on the MCF-7 cell line, with the potential to significantly reduce cell viability. This anticancer efficacy is primarily attributed to D-limonene, the main active component of lemon essential oil.

Notably, all concentrations of the nanoemulsion form of lemon essential oil (LEON) exhibit a stronger effect on breast cancer cells compared to the normal lemon essential oil (LEO). This difference can likely be attributed to the smaller particle size found in the nanoemulsion, enhancing the bioavailability and the overall anticancer activity of the lemon essential oils.

As illustrated in Fig. 33, lemon essential oil displays a dose-dependent cytotoxic effect on MCF-7 cells—as the concentration increases, cell viability declines significantly, confirming its potential antiproliferative capabilities^56^. Reported IC50 values for lemon essential oil range from 84 µg/mL at 24 h to 77 µg/mL at 48 h, which is consistent with the findings of the current study^57^.

Further comparisons in Table 8 and Figs. 34–36 show that lemon essential oil is rich in bioactive compounds such as D-limonene, which have demonstrated significant cytotoxic effects, inhibiting cell viability by up to 98.71%. These compounds are recognized for their role in inducing apoptosis and inhibiting processes like metastasis and angiogenesis^58^. Additionally, citrus limetta essential oil has shown anticancer activity with an IC50 of 47.31 µg/mL, though its efficacy is lower than what was observed in this study, thus reinforcing the potential of citrus-derived essential oils^59^.

The study suggests that nanoemulsions can bolster anticancer activity by facilitating the co-delivery of multiple agents with divergent properties. In the context of MCF-7 cells, lemon essential oil nanoemulsions markedly reduce IC50 values, likely due to their reduced droplet size and negative zeta potential, which enhances cellular uptake. Consistent reductions in cell growth with increasing concentrations corroborate the findings of the current research^60^.

In the present study, the untreated cells served as the negative control to determine the baseline cell viability. A blank nanoemulsion (without garlic essential oil) and a positive cytotoxic control were not included in the experimental design. The primary objective of this work was to compare the cytotoxic effects of free garlic essential oil (GEO) and garlic essential oil-loaded nanoemulsion (GEON) at equivalent concentrations to evaluate whether nanoencapsulation enhances the biological activity of the essential oil. Both formulations were prepared under identical experimental conditions, with the only variable being the encapsulation of the active ingredient, allowing the comparison to directly reflect the influence of the nanoemulsion delivery system on the anticancer activity. Additionally, the excipients used in the nanoemulsion formulation were selected for their well-documented biocompatibility and frequent use in pharmaceutical and food applications at concentrations deemed non-cytotoxic.

In summary, all normal and nano-essential oils examined (both garlic and lemon) exhibit anticancer properties against MCF-7 breast cancer cells, with their effects strongly linked to the principal components of these essential oils. The results indicate that higher concentrations of both normal and nano-essential oils correlate with decreased viability of breast cancer cells.

Statistical analysis reveals a significant difference in anticancer efficacy between normal and nano-essential oils across all tested concentrations, except for the normal and nano garlic essential oils (GEO and GEON) at 100 µg/mL and 250 µg/mL, where no significant difference was detected.

Overall, the findings suggest that normal and nano garlic essential oils (GEO and GEON) display superior anticancer effects, as evidenced by the lowest viability percentages in breast cancer cells. In contrast, both normal and nano lemon essential oils (LEO and LEON) exhibit comparatively lower anticancer efficacy, resulting in higher viability percentages. Additionally, the IC50 values for garlic essential oils are more favorable than those for lemon essential oils.

## Conclusion

The research has highlighted that the effectiveness of garlic and lemon essential oils, along with their nanoemulsions, largely hinges on specific bioactive compounds: trisulfide derivatives in garlic and D-limonene in lemon. These compounds play a crucial role in determining their stability, solubility, and bioavailability, especially in the nanoemulsion form. Variations in their concentrations can result from factors such as the degradation of allicin or differences in extraction methods.

Compared to traditional oils, nanoemulsions have shown enhanced quality. Specifically, garlic oil and its nanoemulsion (GEO and GEON) have demonstrated stronger antioxidant and physicochemical properties than both lemon oil and its nanoemulsion (LEO and LEON).

The findings suggest that both regular and nanoemulsified garlic essential oils are significantly more effective at reducing the viability of breast cancer cells, showcasing a greater anticancer effect. In contrast, conventional and nanoemulsified lemon essential oils display a diminished anticancer effect, as indicated by a higher percentage of viable breast cancer cells.

In summary, these results emphasize the importance of nanoemulsions and indicate a clear need for additional research into their biological effects and safety.

## Experimental section

### Materials and methods

#### Materials

Volatile oils derived from garlic and lemon were procured from the National Research Centre (NRC) in Giza, Egypt. Tween 80 was supplied by (“Nano Fab Technology” / “Company in Giza, Egypt”).

Breast cancer cells (MCF-7) were used in an in vitro cytotoxicity assay and were purchased from Nawah Scientific Research Center via “Holding Company for Biological Products and Vaccines (VACSERA)”, Cairo, Egypt, frozen in liquid nitrogen (-180 °C).

**N.B.** The chemicals mentioned above, including all solvents used, were of the highest purity grade and were purchased from El-Gomhoria for trading chemicals and medical appliances, Mansoura branch, Dakahlia Governorate, Egypt.

#### Methods

##### Separation and analysis of chemical constituents in essential oils using GC/MS

Essential oils were evaluated at the Faculty of Agriculture, Cairo University, Egypt, using a Gas Chromatograph System (HP 6890 Series) connected to an HP 5973 Mass Selective Detector.

A Supelco MDN-5S capillary column (30 m × 0.25 mm, with a film thickness of 0.5 µm) was carefully chosen for its compatibility with the experiment. Helium was the carrier gas, flowing at a constant rate of 1 mL/min. The gas chromatograph oven temperature program started at 45°C, held for 4 min, then increased gradually to 150°C at a rate of 5°C/min, where it was maintained for 5 min. In the end, it reached a temperature of 285°C at a rate of 15°C per minute and maintained that temperature for another four minutes. The injector and detector temperatures were set at 260°C.

A 1.0 μL sample, automatically injected and diluted 1:100 in heptane, was analyzed. Mass spectrometry was performed using electron impact (EI) ionization at 70 eV. The elements were recognized by examining their GC retention durations and decoding.

The related mass spectra. The acquired mass spectra were validated by comparing them with recognized spectroscopic libraries, including the NIST database (National Institute of Standards and Technology) and reference information^61^.

##### Manufacture of nanoemulsion

The nanoemulsion was formed by combining essential oils, water, and a surfactant.

The non-ionic surfactant Tween 80 is frequently utilized to improve the stability of nanoemulsions due to its high solubility in essential oils. It can effectively coalesce with essential oil droplets with some adjustments^62^. In both essential oils, the essential oil-to-surfactant ratio was 1:3 (v/v) (4% essential oil-12% surfactant-84% water), and the two components were combined.

for three hours at 700 rpm.

Nanoemulsions were prepared through a two-step process. Initially, emulsions were formed by combining essential oils (e.g., garlic or lemon) with water and the surfactant Tween 80, followed by homogenization in an ultrasonic bath for 10 min at 260 watts, 50 Hz, and at room temperature. This process was used to ensure proper dispersion. The next phase consisted of continuously mixing the mixture for 3 h at 700 rpm using a magnetic stirrer^63^.

The primary essential oils used in the study were designated as garlic essential oil (GEO) and lemon essential oil (LEO). At the same time, their corresponding nanoemulsion forms were referred to as GEON and LEON, respectively.

##### Characterisation of nanoemulsion

###### Transmission electron microscopy (TEM)

The particle size of nano-essential oils was measured using a JEOL JEM-2100 transmission electron microscope (TEM) at the Electron Microscopy Laboratory, Mansoura University, and College of Agriculture, Egypt.

A drop of the diluted sample was deposited onto copper grids with a carbon support film and allowed to air-dry at room temperature.

Imaging was performed with a high-resolution FEI Tecnai G2-12 Spirit Biotwin TEM system. A minimum of 10 locations on the transmission electron microscope grid were examined. The optimal nanoparticle concentration was identified using a methodology based on benchmarks established by a recognized standards authority, the National Institute of Standards and Technology (NIST). Image analysis was conducted using the freely available software ImageJ, which can be accessed online. The dimensions of the areas covered by the oval selection tools were determined once they were modified to align with the scale bar in the TEM images^62,63^.

###### Zeta potential

The Zeta potential of the nano-essential oils was measured by assessing their electrophoretic mobility in an electric field after a 100-fold dilution in ultrapure water. The analysis was performed at 25°C using a Malvern Zetasizer Nano-ZS90.

Measurements in the electrophoretic cell utilized a viscosity (cP) of 0.8872,

a dispersant RI of 1.330, and a potential of ± 150 mV. Data analysis was performed using.

The Helmholtz-Smoluchowski approximation^64^. Each measurement was repeated three times at the Electron Microscopy Laboratory at Mansoura University, Faculty of Agriculture, Egypt.

##### Chemical characteristics of normal and nano essential oils

###### Acid number

The acid value refers to the amount of potassium hydroxide in milligrams required to neutralize the free fatty acids in one gram of fat. Moreover, since free fatty acids are generally generated during the degradation of triglycerides, they act as an indicator of rancidity. The acid value was calculated using the formula:

$${\text{Acid Value }} = \, \left( {{\text{Normality of NaOH }} \times {56}.{1 } \times {\text{ Titer Value}}} \right)/{\text{Sample Weight }}\left( {\mathrm{g}} \right).$$

The samples’ acid value (AV) was determined following the **AOCS** guidelines established in^65^.

###### Peroxide number

Oxidative properties can lead to rancidity. Oxidative rancidity occurs when fats or oils.

are exposed to oxygen and create peroxides.

The peroxide value of the oil serves as an indicator of its peroxide content^66^.

The appropriate unit for peroxide value (PV) is:

Milliequivalents of active oxygen per kilogram of oil (meq O₂/kg).

This unit measures the amount of peroxide (primary oxidation products) present in fats or oils, indicating the extent of oxidative rancidity.

The peroxide value (PV) was calculated using the formula:

$${\text{PV }} = \, \left( {\left( {{\text{Sample Titration }}{-}{\text{ Blank Titration}}} \right) \, \times {\text{ Normality of Na}}_{2} {\mathrm{S}}_{2} {\mathrm{O}}_{3} \, \times { 1}000} \right)/{\text{Weight of the oil sample}}.$$

The standardized procedures outlined assessed the peroxide values^65^.

##### Free radical scavenging assay (DPPH) of normal and nano essential oils

The frequently used DPPH reaction relies on the ability of antioxidant molecules to act as a hydrogen donor to 2,2-diphenyl-1-picrylhydrazyl (DPPH), resulting in its conversion to an inactive form.

Using a validated DPPH radical scavenging assay, the capacity of both regular and nano essential oils to neutralize the “stable” free radical DPPH was evaluated, following the procedures outlined in^67^*.*

The percentage (%) of DPPH free radical Inhibition was determined using.

The following calculation:

$$\% {\text{ inhibition }} = \, \left( {\left( {{\text{A control }}{-}{\text{ A test}}} \right)/{\text{ A control}}} \right) \, *{ 1}00$$

A blank: uptake of a blank specimen.

A specimen: Uptake of specimen solution.

All experiments were conducted in three repetitions.

##### Biological effect of normal and nano essential oils on breast cancer cells

The impact of varying concentrations of standard and nano-essential oils on the viability of breast cancer cells (MCF-7) was assessed using the MTT colorimetric technique. In summary, the cells were seeded into a 96-well culture plate at a density of 10,000 cells per well and incubated for 48 h with different concentrations of all normal and nano essential oils. The cells received treatment with standard and nano essential oils at concentrations of 50, 100, 150, 200, and 250 μg/mL. Once the culture media were removed, the cells were exposed to MTT solution (5 mg/mL in PBS) for three hours, and 50 µL of DMSO (Sigma) was added to dissolve the resultant formazan. The absorbance was recorded using a plate reader (BioTek ELx800) at 540 nm instead of 570 nm^68^.

The term “IC50” denotes the drug concentration required to reduce the count of viable cells by 50% in vitro, according to each cytotoxicity curve^68^. They explained that the true IC50 values ​​of valuable and promising glioblastoma treatment compounds may be missed due to overestimation or underestimation. Therefore, the values ​​were measured at 540 nm instead of 570 nm.

The cytotoxicity assay methods were developed by adhering to the standard operating procedure for cell culture and harvesting. The IC50 concentration was determined based on the density of the cell stock culture used to establish an assay^69^.

##### Statistical analysis

The collected data are presented as means ± S.D., and statistical analysis was conducted using one-way analysis of variance (ANOVA). The means between groups were compared using the (LSD) statistical test at a 5% significance level, employing the computer program (Costate) in accordance with Gomez and Gomez (1984)^70^.

## Data Availability

All data used are mentioned in the paper. The datasets used and/or analyzed during the current study are available from the corresponding author on reasonable request.
